# Reduction of bitter taste receptor gene family in folivorous colobine primates relative to omnivorous cercopithecine primates

**DOI:** 10.1007/s10329-024-01124-w

**Published:** 2024-04-11

**Authors:** Min Hou, Muhammad Shoaib Akhtar, Masahiro Hayashi, Ryuichi Ashino, Akiko Matsumoto-Oda, Takashi Hayakawa, Takafumi Ishida, Amanda D. Melin, Hiroo Imai, Shoji Kawamura

**Affiliations:** 1 Department of Integrated Biosciences, Graduate School of Frontier Sciences, The University of Tokyo, Bioscience BLDG Room 502, 5-1-5 Kashiwanoha, Kashiwa, Chiba 277-8562 Japan; 2 Graduate School of Tourism Sciences, University of the Ryukyus, Nishihara, Okinawa Japan; 3 Faculty of Environmental Earth Science, Hokkaido University, Sapporo, Hokkaido Japan; 4 Japan Monkey Centre, Inuyama, Aichi Japan; 5 Department of Biological Sciences, Graduate School of Science, The University of Tokyo, Tokyo, Japan; 6 Department of Anthropology and Archaeology, University of Calgary, Alberta, Canada; 7 Department of Medical Genetics, University of Calgary, Alberta, Canada; 8 Alberta Children’s Hospital Research Institute, University of Calgary, Alberta, Canada; 9 Molecular Biology Section, Center for the Evolutionary Origins of Human Behavior, Kyoto University, Kyoto, Aichi Japan

**Keywords:** TAS2R, Cercopithecines, Colobines, Targeted capture, Phylogeny-based probe design, Birth and death evolution

## Abstract

Bitter taste perception is important in preventing animals from ingesting potentially toxic compounds. Whole-genome assembly (WGA) data have revealed that bitter taste receptor genes (*TAS2R*s) comprise a multigene family with dozens of intact and disrupted genes in primates. However, publicly available WGA data are often incomplete, especially for multigene families. In this study, we employed a targeted capture (TC) approach specifically probing *TAS2R*s for ten species of cercopithecid primates with diverse diets, including eight omnivorous cercopithecine species and two folivorous colobine species. We designed RNA probes for all *TAS2R*s that we modeled to be intact in the common ancestor of cercopithecids (“ancestral-cercopithecid *TAS2R* gene set”). The TC was followed by short-read and high-depth massive-parallel sequencing. TC retrieved more intact *TAS2R* genes than found in WGA databases. We confirmed a large number of gene “births” at the common ancestor of cercopithecids and found that the colobine common ancestor and the cercopithecine common ancestor had contrasting trajectories: four gene “deaths” and three gene births, respectively. The number of intact *TAS2R* genes was markedly reduced in colobines (25–28 detected via TC and 20–26 detected via WGA analysis) as compared with cercopithecines (27–36 via TC and 19–30 via WGA). Birth or death events occurred at almost every phylogenetic-tree branch, making the composition of intact genes variable among species. These results show that evolutionary change in intact *TAS2R* genes is a complex process, refute a simple general prediction that herbivory favors more *TAS2R* genes, and have implications for understanding dietary adaptations and the evolution of detoxification abilities.

**Supplementary Information:**

The online version contains supplementary material available at 10.1007/s10329-024-01124-w.

## Introduction

Gustation (taste perception) is a primary sense of vertebrates, conveying tastant chemical information from the oral cavity to the brain to regulate ingestion (Miura and Barlow 2010). Among the five basic taste senses, bitterness, sweetness, umami, sourness, and saltiness (Kinnamon and Cummings 1992; Lindemann 1996), bitter taste perception is considered to help animals to detect potentially toxic compounds and to prevent animals from ingesting them. Bitter taste perception is mediated by a group of seven-transmembrane G-protein-coupled receptors (GPCRs) known as TAS2Rs (also designated as T2Rs) (Adler et al. 2000). Mammals typically possess dozens of *TAS2R* genes, which are intronless, typically ~900 base pairs (bp) long and part of the same multigene family (Nei et al. 2008). The multiple *TAS2R* genes are spread over several chromosomes and are tandemly clustered in each of those chromosomes.

With the advent of massive parallel sequencing [next-generation sequencing (NGS)] technologies, the *TAS2R* sequences of various species have been accessible through public whole genome assembly (WGA) databases. In the human reference genome database, there are 26 intact and 10 disrupted (pseudogenized) *TAS2R* genes found (Hayakawa et al. 2014). The number of putative *TAS2R* genes in vertebrate genomes vary among species from a few to approximately 50 (Li and Zhang 2014). A comparative study of *TAS2R* genes has been of ongoing research interest given their potential to provide insight into the genetic basis of dietary adaptations and feeding strategies (Dong et al. 2009; Hayakawa et al. 2014; Li and Zhang 2014; Liu et al. 2016; Shi and Zhang 2006).

Glendinning (1994) showed that carnivorous mammals, which rarely encounter bitter and potentially poisonous foods, had a low bitter threshold (i.e., high bitter sensitivity) and low tolerance to ingest toxic compounds, while herbivorous mammals, especially browsers, which commonly encounter bitter and potentially poisonous foods, had a high bitter threshold (i.e., low bitter sensitivity) and high tolerance to ingest toxic compounds. Omnivorous mammals were positioned between the two groups (Glendinning 1994). Based on this observation, we would hypothesize that herbivorous animals have fewer functional *TAS2R* genes than carnivorous or omnivorous animals, perhaps because they have evolved dietary adaptations to tolerate toxic foods and need not avoid bitter foods. For example, herbivorous ruminants have evolved foregut fermentation by microbes and have higher tolerance for toxic compounds (Freeland and Janzen 1974). However, a later study of 54 vertebrate WGA databases (including 41 mammals, four birds, two reptiles, one amphibian, and six fishes) reported that the number of intact *TAS2R* genes positively correlated with herbivory, i.e., the fraction of plants in their diet (Li and Zhang 2014). This suggests that herbivores may need to recognize a larger number of bitter compounds than carnivores and omnivores to select the type of plants they eat (Li and Zhang 2014). Nevertheless, this would not necessarily refute the prediction based on Glendinning’s (1994) findings, because effects of ecological factors on *TAS2R* gene evolution could be complex. As Li and Zhang (2014) suggest, it is imperative to examine a diverse group of species when testing the potential impact of an ecological factor on taste receptor gene evolution.

Primate species belonging to the family Cercopithecidae are widely distributed among diverse habitats in sub-Saharan and northernmost Africa and southern, south-eastern to eastern Asia. They are composed of two subfamilies Cercopithecinae (including macaques, baboons, and guenons) and Colobinae (including colobuses, langurs, lutungs, and surilis). While cercopithecine monkeys are omnivorous with diets including fruits, leaves, seeds, buds, mushrooms, insects, spiders, and smaller vertebrates, the colobine monkeys are specialized for folivory supplemented with flowers, fruits, and occasional insects (Fleagle 2013). Colobine monkeys differ from all other primates in having a foregut-fermentation digestive system (Bauchop and Martucci 1968). To aid in digestion, especially of leaves that are difficult to digest, they possess three or four chambers in their forestomach. Although plant tissues contain more toxic compounds compared with animal tissues, foregut fermenters use symbiont microorganisms (i.e., protists and prokaryotes) to detoxify plant compounds before reaching the intestine, where toxins can be absorbed. Furthermore, the digestive efficiencies of plant fiber are significantly higher in foregut fermenters compared with hindgut fermenters (Edwards and Ullrey 1999). The foregut bacteria are digested by the specialized lysozyme that evolved to be active at low pH, resistant to protease, and expressed in the stomach to recover nutrients (Messier and Stewart 1997). The bacterial RNA molecules are degraded in the small intestine by the specialized RNASE1B to recycle nitrogen efficiently (Guevara et al. 2021a, b; Zhang et al. 2002).

Comparison of the *TAS2R* gene repertoire among phylogenetically close but ecologically diverse species would facilitate our understanding of ecological (dietary) effect on evolutionary diversity and adaptation of *TAS2R* genes. Thus, cercopithecid monkeys would be a suitable animal group for testing the potential impact of ecological factors on *TAS2R* gene evolution. Despite their importance, cercopithecid monkeys have been underrepresented in previous studies of *TAS2R* gene repertoire: one cercopithecid species (rhesus macaque) in Li and Zhang (2014) and three cercopithecid species (rhesus macaque, crab-eating macaque, and anubis baboon) in Hayakawa et al. (2014). The *TAS2R* gene repertoire has not yet been investigated for any colobine species.

In addition, dependence of studies of the *TAS2R* gene repertoire on publicly available WGA databases could be potentially problematic due to their generally low sequencing depth and inherent incompleteness especially for multigene families, such as *TAS2R*s. In this study, we applied the targeted capture method and the short-read massive parallel sequencing to achieve high-depth sequencing to reveal *TAS2R* gene repertoire for five genera, eight species from cercopithecines and two genera, two species from colobines. Combining data from currently available WGA databases, we clarify evolutionary trajectory of *TAS2R* gene “births” and “deaths” compared between cercopithecines and colobines and among species within each subfamily. We reveal whether folivorous colobine species have *TAS2R* gene repertoires that differ from omnivorous cercopithecine species and discuss implications for the effect of ecological factors on shaping the evolution of the *TAS2R* gene repertoire.

## Materials and methods

### Genomic DNA samples

**Fig. 1:**
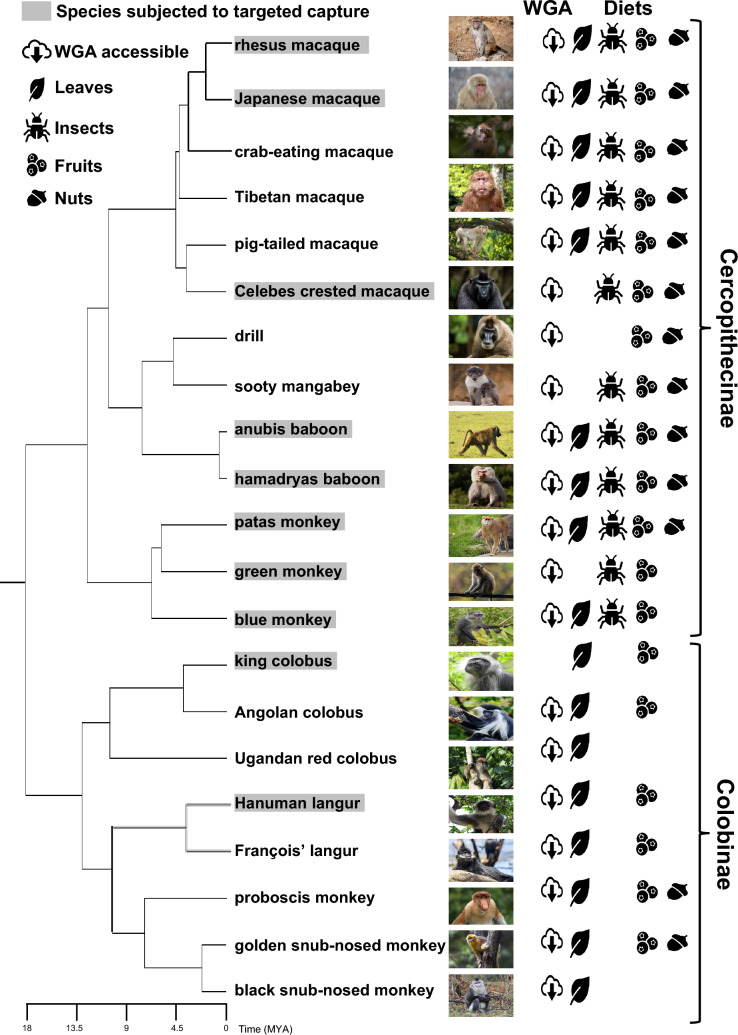
The phylogeny and primary diets of cercopithecid primates which were considered in this study. The phylogenetic relationship and divergence times were generated using TimeTree (Kumar et al. 2017) and modified based on literature (Kuderna et al. 2023; Li et al. 2009; Perelman et al. 2011). The species subjected to the targeted capture are highlighted with gray shadow. The other species were considered for the mapping references and subsequent analyses. Species for which public WGA databases are accessible are given with cloud symbol. Primary diets are symbolized for leaves, insects, fruits, and nuts (Fleagle 2013). Licensed photographs were obtained from Shutterstock (https://www.shutterstock.com) and license-free photographs were from Pixabay (https://pixabay.com). For detailed credit information, see Table S11

We targeted and captured *TAS2R* genes from genomic DNA samples from ten study species (one individual each) in the family Cercopithecidae (Table 1, Fig. 1). Eight species were from the subfamily Cercopithecinae: three species of the genus *Macaca* (*Macaca mulatta*, *Macaca fuscata*, and *Macaca nigra*), two species of the genus *Papio* (*Papio anubis* and *Papio hamadryas*), three species of the tribe Cercopithecini (*Erythrocebus patas*, *Chlorocebus sabaeus*, and *Cercopithecus mitis*). Two species were from the subfamily Colobinae: *Colobus polykomos* and *Semnopiethecus entellus*. The genetic materials from Primate Research Institute, Kyoto University, Japan, were obtained through their Cooperative Research Program (approval numbers 2012-A-11, 2020-A-27, and 2021-A-25). Semen of *P. anubis* was collected opportunistically by A.M.O. at the time a wild troop census in Kenya and transported to Japan through an export permit no. 0831553 issued by Kenya Wildlife Service, Republic of Kenya. The cellular sources of *Erythrocebus patas*, *Cercopithecus mitis*, *Colobus polykomos*, and *Semnopithecus entellus* were obtained by T. I. in 1983, 1984, 1988, and 1984, respectively, and cultured and transformed by T. I. (Ishida and Yamamoto 1987).

**Table 1:** Sources of cercopithecid samples examined

Common name	Species name	Abbreviation	Sex	Cell/tissue sources	Provided from
Rhesus macaque	*Macaca mulatta*	Mmul	Male	Blood	Primate Research Institute, Kyoto University
Japanese macaque	*Macaca fuscata*	Mfus	Male	Blood	Primate Research Institute, Kyoto University
Celebes crested macaque	*Macaca nigra*	Mnig	Male	Muscles	Primate Research Institute, Kyoto University
Anubis baboon	*Papio anubis*	Panu	Male	Semen	Akiko Matsumoto, University of the Ryukyus
Hamadryas baboon	*Papio hamadryas*	Pham	Female	Blood	Primate Research Institute, Kyoto University
Patas monkey	*Erythrocebus patas*	Epat	Male	Epstein–Barr virus-transformed B cells	Takafumi Ishida, The University of Tokyo
Green monkey	*Chlorocebus sabaeus*	Csab	Male	Blood	Primate Research Institute, Kyoto University
Blue monkey	*Cercopithecus mitis*	Cmit	Female	Endogenous herpes virus-transformed B cell line	Takafumi Ishida, The University of Tokyo
King colobus	*Colobus polykomos*	Cpol	Female	Epstein–Barr virus-transformed B cells	Takafumi Ishida, The University of Tokyo
Hanuman langur	*Semnopithecus entellus*	Sent	Male	Epstein–Barr virus-transformed B cells	Takafumi Ishida, The University of Tokyo

### *TAS2R* sequence source information for designing probe

We aim to examine *TAS2R* genes, which were intact in the common ancestor of cercopithecids (designated as “ancestral-cercopithecid *TAS2R* genes”). We regarded such *TAS2R* genes as those that were intact at least one species among the five cercopithecid WGA databases available at the time we started this project (three cercopithecines: *Macaca mulatta* Mmul_8.0.1 (Zimin et al. 2014), *M. fascicularis* Macaca_fascicularis_5.0, and *Cercocebus atys* Caty_1.0 (Palesch et al. 2018) and two colobines: *Colobus angolensis* Cang.pa_1.0 and *Rhinopithecus roxellana* Rrox_v1 (Zhou et al. 2014) (Table S1-1). We conducted a Basic Local Alignment Search Tool (BLAST) search (Johnson et al. 2008; NCBI Resource Coordinators 2016) to these databases for *TAS2R* genes that had been identified by Hayakawa et al. (2014), who studied humans, various other primates, and nonprimate mammals. There were 31 such genes (Table S2-1). Hayakawa et al. (2014) examined three cercopithecid species (rhesus macaque, crab-eating macaque, and anubis baboon) which were available at the time of their study and identified one additional gene, *TAS2R418*, although the gene was disrupted in all these three species. Their assignment as an intact gene at the cercopithecid ancestor was because the disruptive mutations were different between the macaques and the baboon. However, we excluded this gene from our assignment because its sequence was disrupted in not only in our choice of the three cercopithecine WGA databases but also in the two colobine ones.

As the source of probe DNA sequences (Table S1-1) for cercopithecine *TAS2R* genes, we chose Mmul_8.0.1 first, Macaca_fascicularis_5.0 second, and Caty_1.0 third. For the colobine *TAS2R* genes, we first chose Cang.pa_1.0 and second Rrox_v1. If a gene of interest was absent in the WGA database chosen, we searched the lower priority WGA databases for an intact form of the gene. If a gene of interest was disrupted (indicated with a suffix “P” in Table S2-1) or “truncated” (suffix “T”) in the WGA database chosen, we took it as a probe sequence and searched the next WGA databases for an intact form of the gene to add. If a gene of interest was duplicated or multiplicated, including intact genes in the WGA database chosen, we took all these paralogous genes as probe sequences. If duplicated/multiplicated genes were all disrupted or truncated genes, we took the disrupted/truncated ones and searched for the next WGA databases for an intact form of the gene to add (e.g., *TAS2R1* in Angolan colobus Cang.pa_1.0 in Table S2-1). For each probe sequence, 100-nucleotides (nt) upstream and downstream flanking regions were included in addition to the entire coding region (approximately 900 bp) (Table S2-1). The probe sets from cercopithecine and from colobine databases were mixed and used in the targeted capture for all the samples.

### Neutral reference sequence source information for designing probes

As a control reference to evaluate gene copy number and selective neutrality, a previous study (Akhtar et al. 2022) selected 85 single-locus nonprotein-coding sequences (82 autosomal and three X-nonpseudoautosomal) from the human reference genome hg19 assembly using the Neutral Region Explorer (Arbiza et al. 2012). In brief, the selected regions were without repeat sequences, with a length longer than 1.0 kb, with distance to the nearest gene longer than 0.2 centimorgan (cM) in autosomes or 0.1 cM in the X chromosome, and with a minimum recombination rate of 0.9 cM/Mb under the Neutral Region Explorer. We conducted a BLAST search using these human sequences as queries for the rhesus macaque Mmul_8.0.1 and Angolan colobus Cang.pa_1.0 WGA databases. We took the sequences with a reasonably high similarity (higher than 90%) between human and cercopithecid nonprotein coding regions. When present, there was only one such region detected for each sequence. Although we have not confirmed their orthology to the human sequences by reconstructing the phylogenetic tree, there were no protein-coding genes among them, and these were with a length longer than 1.0 kb. We regarded these regions usable as neutral references in this study. Among the 85 regions, 83 and 79 regions were identified from Mmul_8.0.1 and Cang.pa_1.0 WGA databases, respectively, and used as probes (Table S2-2).

### Probe synthesis, targeted capture, and NGS

Probe synthesis was outsourced to the Biodiscovery, LLC (Ann Arbor, MI) as the in-solution biotinylated RNA baits (myBaits^®^) (Gnirke et al. 2009). Each probe was 120-nt length and was overlapped 60 nt with adjacent probes (i.e., 2× tiling). Construction of the DNA sequencing libraries and targeted capture were performed using myBaits^®^ kit v5 following the manufacturer’s protocol (myBaits^®^ user manual version 5.02). Construction of DNA sequencing library, targeted capture, and NGS were conducted by the Centre for Health Genomics and Informatics and UCDNA Service, University of Calgary, Canada. DNA sequencing libraries were constructed by fragmenting genomic DNA into ~500 bp. Biotinylated RNA baits were introduced and allowed to hybridize to targets at 65 °C. Bait-target hybrids were then pulled out of the solution with streptavidin-coated magnetic beads. The beads were stringently washed at 65 °C to remove nonhybridized and nonspecifically-hybridized molecules. These conditions of hybridization and washing are reported to allow ~10% mismatch between baits and targets according to the manufacturer’s protocol (myBaits^®^ user manual version 5.02). The captured DNA library was released from the beads and amplified by polymerase chain reaction (PCR) targeting the adapter sequence. The massive parallel sequencer used in this study was the Illumina NextSeq with 300 cycle and 150-bp paired end sequencing.

### Quality control of NGS reads

We used the PRINSEQ tool for quality control of raw NGS reads (Schmieder and Edwards 2011). For the FASTQ-formatted reads with the base quality score emitted by the Illumina sequencer at each nucleotide position, any nucleotide position with quality score below 20 at either edge of a read was trimmed away. Any read with a mean quality score less than 20 and any read shorter than 10 nucleotides were also filtered out.

### Reference sequences for the first-round mapping of NGS reads

For mapping the NGS short reads to reference sequences, we aimed to choose the WGA databases of the phylogenetically closest species to the study species (Table S1-2). At the time we did the mapping, WGA databases of 23 cercopithecid species were publicly accessible. Fifteen species were from Cercopithecinae: six species of *Macaca* (*Macaca mulatta*, *Macaca fuscata*, *Macaca fascicularis*, *Macaca thibetana*, *Macaca nemestrina*, and *Macaca nigra*), *Mandrillus leucophaeus*, *Cercocebus atys*, two species of *Papio* (*Papio anubis* and *Papio hamadryas*), *Erythrocebus patas*, two species of *Chlorocebus* (*Chlorocebus aethiops* and *Chlorocebus sabaeus*), and two species of *Cercopithecus* (*Cercopithecus albogularis* and *Cercopithecus mitis*). Eight species were from Colobinae: two species of *Colobus* (*Colobus angolensis palliates* and *Colobus guereza*), *Piliocolobus tephrosceles*, *Semnopiethecus entellus*, *Trachypithecus francoisi*, *Nasalis larvatus*, and two species of *Rhinopithecus* (*Rhinopithecus bieti* and *Rhinopithecus roxellana*) (Jayakumar et al. 2021; Liu et al. 2020; Palesch et al. 2018; Sène et al. 2021; Shao et al. 2023; Simons et al. 2019; Wall et al. 2022; Wang et al. 2019; Warren et al. 2020; Zhang et al. 2022; Zhou et al. 2023).

In addition to the 31 probe-designed *TAS2R* genes, we examined 22 other *TAS2R* genes that were absent or disrupted in all these cercopithecid WGA databases but intact in WGA databases of some noncercopithecid primate (Table S3). If the cercopithecid genes were present as a disrupted form, they were used for references. If the cercopithecid genes were truncated or absent, genes from closer outgroup species were used.

### Mapping, assemblage, and inference of gene duplications

Our mapping procedure is described below and is illustrated in Fig. S1.

1. First-round mapping

We mapped the filtered reads using the BWA-MEM Tool v. 0.7.17 to the references (Table S3), which were assigned to each *TAS2R* gene of each study species for the first-round mapping. The tool is specialized for mapping highly similar reads on a reference (Zheng-Bradley et al. 2017), which is the case with *TAS2R* genes because of the presence of similar paralogous genes. After the mapping, by applying the MarkDuplicates of the Genome Analysis Toolkit (GATK) (DePristo et al. 2011; McKenna et al. 2010; Van der Auwera et al. 2013), we identified the read pairs that were likely to have originated from duplicates of the same original DNA fragments through artefactual processes, such as PCR amplification. Only a single read pair within each set of duplicates was used for the subsequent analyses.

The HaplotypeCaller of GATK (4.2.5.0) was used to assemble the mapped reads, to find insertion/deletion (indel) sites and single-nucleotide polymorphism (SNP) sites, and to determine haplotype phase among these polymorphic sites based on their physical linkage in a sequencing read. We defined an indel site as the site where insertion or deletion of a nucleotide was detected among sequencing reads relative to the reference sequence. We defined an SNP site as the site where two or more nucleotide kinds were present among sequencing reads. We did not count nucleotide sites as SNPs where only one nucleotide kind was detected among reads but the nucleotide kind was different from the one in the reference at the site. Two sequences were generated as putative haplotypes by bcftools consensus v. 1.7. If no read is mapped to a region in a reference sequence, the HaplotypeCaller generates chimeras consisting of the sample’s own sequences for the mapped region and the reference sequences for the unmapped region.

2. Second-round mapping

Less mismatch is expected between a sequence generated from the first-round mapping and a sequencing read from a gene/genome region. To improve the proportion of mapped regions, we conducted the second-round mapping using the chimeric sequences generated from the first-round mapping. For the second-round mapping references, we used one of the two putative haplotype sequences bearing nucleotides from the sample but not from the reference at SNP sites. Mapping was conducted as in the first mapping. Variant calling for each site was conducted with samtools mpileup v. 1.7 (Li 2011) to identify unmapped regions. Two sequences were generated as putative haplotypes by bcftools consensus v. 1.7. In the generated sequences, we exchanged nucleotides in these unmapped regions for “N”s. We evaluated the sequencing depth of the *TAS2R* genes and of the neutral references using the samtools bedcov (Li 2011).

3. Third-round mapping

For the third-round mapping, we used the sequences generated from the second-round mapping including “N” regions. There, we used one of the two putative haplotype sequences bearing a smaller number of deletions relative to the reference sequence. We regarded genes/genome regions with low depth (< ten per site) after the second-round mapping as being absent and excluded them from the third-round mapping to improve its specificity. Mapping was conducted as in the first mapping. The HaplotypeCaller was used to assemble the mapped reads and determine haplotype phase among SNPs and indels as in the first mapping. We further refined variant calls using VariantFiltration of GATK. Any variant sites with a genotype quality score less than 20 were filtered out. Two sequences were generated as putative haplotypes by bcftools consensus v. 1.7. The International Union of Pure and Applied Chemistry (IUPAC) degenerate nucleotide codes were used to designate unphased SNP sites with more than one nucleotide kinds. We removed the “N” sites from the generated sequences, which makes them consisting of only the sample’s own sequences.

After the third-round mapping, we evaluated again the sequencing depth of the *TAS2R* genes (Table S4-1) and of the neutral references (Table S4-2) using the samtools bedcov (Li 2011). We regarded the *TAS2R* genes and the autosomal neutral references (NR-As) with sequencing depth lower than half of the minimum of lower whisker in boxplots (not shown) of the sequencing depth as being possibly absent and excluded from further analyses. We also excluded the *TAS2R* genes as possibly absent of which the length of mapped sequence was less than 750 bp (see the section “Classification of *TAS2R* sequences into intact and disrupted types”). We excluded the neutral references shorter than 1000 bp from the subsequent analyses. We also excluded from further analyses the NR-As with sequencing depth 1.5 times higher than the maximum of upper whisker in the boxplots as being potentially duplicated in at least one allele.

Boxplots of sequencing depths of *TAS2R* genes and of neutral references after these treatments are shown in Fig. S2. Using the same sequence data set, we evaluated the number of SNP sites (not including indels) per 1000 bp (SNP density) of the *TAS2R* genes (Table S5-1) and of the NR-As (Table S5-2) using vcftools v 0.1.13 (Danecek et al. 2011). Boxplots of the SNP density of *TAS2R* genes and NR-As are shown in Fig. S3. We regarded the *TAS2R* genes as single-locus genes, of which both sequencing depth (Fig. S2) and SNP density (Fig. S3) were below the maximum of the upper whisker of the corresponding boxplots. We generated two putative haplotype sequences for these genes by the HaplotypeCaller after the third mapping and regarded the two sequences, designated as H1 and H2, as two alleles of these genes.

4. Fourth-round mapping

To separate sequencing reads into paralogous *TAS2R* genes, we regarded the *TAS2R* genes as potentially duplicated or multiplicated when either sequencing depth (Fig. S2) or SNP density (Fig. S3) was above the maximum of the upper whisker (“upper outlier”) of the corresponding boxplots. To tease apart the sequencing reads into potentially paralogous genes, we generated two putative haplotype sequences (H1 and H2) using the HaplotypeCaller after the third-round mapping and used both of the two putative haplotype sequences as the references of the fourth-round mapping. The genes were classified into the following four categories (Table 2)

**Table 2:** Categorization of potentially duplicated/multiplicated genes inferred after the third-round mapping in the ancestral-cercopithecid *TAS2R* gene set

Species	(1) Not separable to single-locus genes at the fourth mapping	(2) Separated to single-locus genes at the fourth mapping	(3) Not separable to single-locus genes at the fifth mapping	(4) With high SNP density but not with high sequencing depth at the fourth or fifth mapping
Rhesus macaque		*414 Ma-1, 414 Ma-2* *415 Ma-1*, *415 Ma-2*		*413*
Japanese macaque		*414 Ma-1, 414 Ma-2* *415 Ma-1*, *415 Ma-2*		*20*
Celebes crested macaque	–	–	*414 Ma-2* *415 Ma-2*	*414 Ma-1* *415 Ma-1*
Anubis baboon	–	*10 Pa-1, 10 Pa-2* *50 Pa-1, 50 Pa-2* *413 Pa-1, 413 Pa-2* *414 Pa-1, 414 Pa-2*	*415 Pa-1, 415 Pa-2*	
Hamadryas baboon		*10 Pa-1, 10 Pa-2* *419 Pa-2*		*418* *419 Pa-1*
Patas monkey	*415*	–	*414Er-1–2*	*403* *411* *414Er-1–1* *419*
Green monkey		*415Ch-1, 415Ch-2*	*414Ch-1–1, 414Ch-1–2*	
Blue monkey	*414*	*415Ce-2*		*415Ce-1*
King colobus		–	–	*42* *412*
Hanuman langur	–	*60Se-1, 60Se-2*	*14Se-1, 14Se-2*	

.

1. *TAS2R* genes not separable to single-locus genes at the fourth mapping: If the same reads were mapped to the two references, H1 and H2, from a source gene and if the proportion of such region was more than roughly 10% of a reference length in the Integrative Genomics Viewer (IGV) (Robinson et al. 2011), we regarded the source gene sequence at the third mapping (i.e., H1 + H2 congregated sequence) as mixture of reads of paralogous and allelic origins, which were not separable to single-locus genes. We regarded the depth and SNP density results at the third-round mapping as the final data for this congregated gene. When evaluating open-reading frame (ORF) and seven transmembrane (7-TM) domain (see the section “Classification of *TAS2R* sequences into intact and disrupted types”), we tentatively used their H1 and H2 sequences. We displaced to category 4 the genes in which the SNP density was in the upper outlier but the sequencing depth was below the maximum of the upper whisker at the third mapping.
2. *TAS2R* genes separated to single-locus genes at the fourth mapping: If the proportion of the same reads mapped to the two references, H1 and H2, from a source gene was less than roughly 10% of a reference length in the IGV, we redesignated the H1 and H2 with suffix “− 1” and “− 2”, respectively, as paralogous genes. If both sequencing depth and SNP density after the fourth mapping were below the maximum of the upper whisker, we regarded such a gene as a single-locus gene. We further generated two haplotype sequences from it by the HaplotypeCaller after the fourth mapping and designated the two sequences with H1 and H2 as its two alleles.
3. *TAS2R* genes not separable to single-locus genes at the fifth mapping: The same situation with category 2, but, if sequencing depth and/or SNP density after the fourth mapping were above the maximum of the upper whisker, we regarded such a gene (with suffix “− 1” or “− 2”) still as potentially duplicated or multiplicated. We further generated two putative haplotype sequences (H1 and H2) by the HaplotypeCaller after the fourth-round mapping and used both two putative haplotype sequences as the references of the fifth-round mapping. The *TAS2R* genes of category 3 turned out to fall in category 1 in the fifth-round mapping. We regarded the source gene sequence at the fourth mapping (i.e., H1 + H2 congregated sequence) as mixture of reads of paralogous and allelic origins, which were not separable to a single-locus gene. We regarded the depth and SNP density results at the fourth-round mapping as the final data for this congregated gene. Only for evaluating ORF and 7-TM domain (see the section “Classification of *TAS2R* sequences into intact and disrupted types”), we tentatively used their H1 and H2 sequences. We displaced the genes to category 4 of which the SNP density was in the upper outlier, but the sequencing depth was below the maximum of the upper whisker at the fourth mapping.
4. The *TAS2R* genes with high SNP density but not with high sequencing depth at the fourth or fifth mapping: This category of *TAS2R* genes were displaced from category 1 or 3. The reason for this high SNP density remains to be elucidated. We waived decision on whether these genes were duplicated/multiplicated or single-locus.

**Fig. 2:**
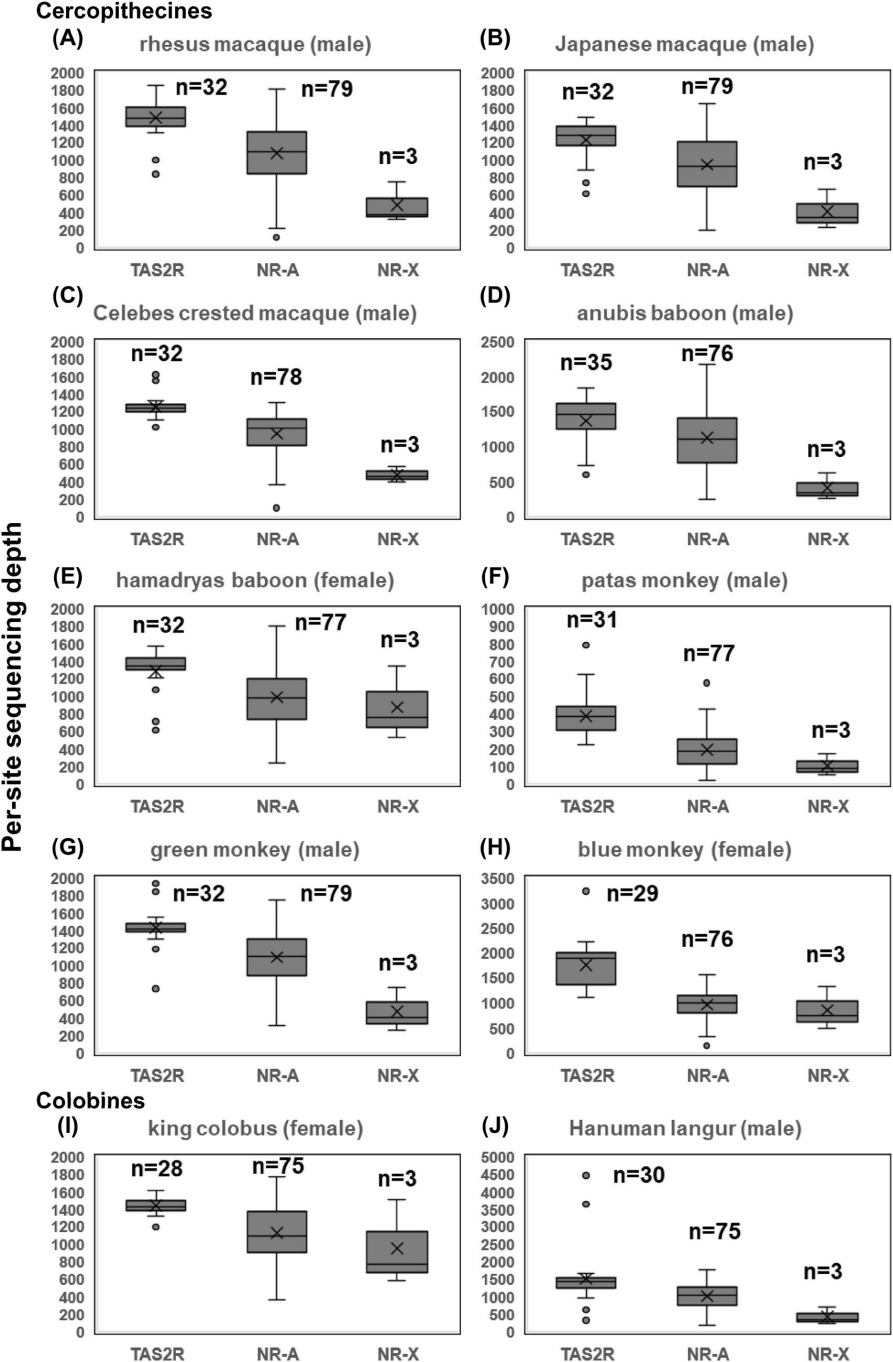
The range of the final per-site sequencing depth of the ancestral-cercopithecid *TAS2R* genes, that of autosomal neutral references (NR-A) and that of the X-chromosomal neutral references (NR-Xs) in each cercopithecid study species, depicted by boxplots. **A**–**H** are cercopithecine species: **A** rhesus macaque, **B** Japanese macaque, **C** Celebes crested macaque, **D** anubis baboon, **E** hamadryas baboon, **F** patas monkey, **G** green monkey, and **H** blue monkey. **I** and **J** are colobine species: **I** king colobus and **J** Hanuman langur. The medians are indicated as the horizontal line in the box. The means are indicated as the symbol “X.” The upper whisker value represents the largest within 1.5 times interquartile range above the third quartile, and the lower whisker value represents the smallest within 1.5 times interquartile range below the first quartile. The outliers are indicated by dots. The median was included in the calculation to determine quartile values. The number of data points are indicated with *n*

**Fig. 3:**
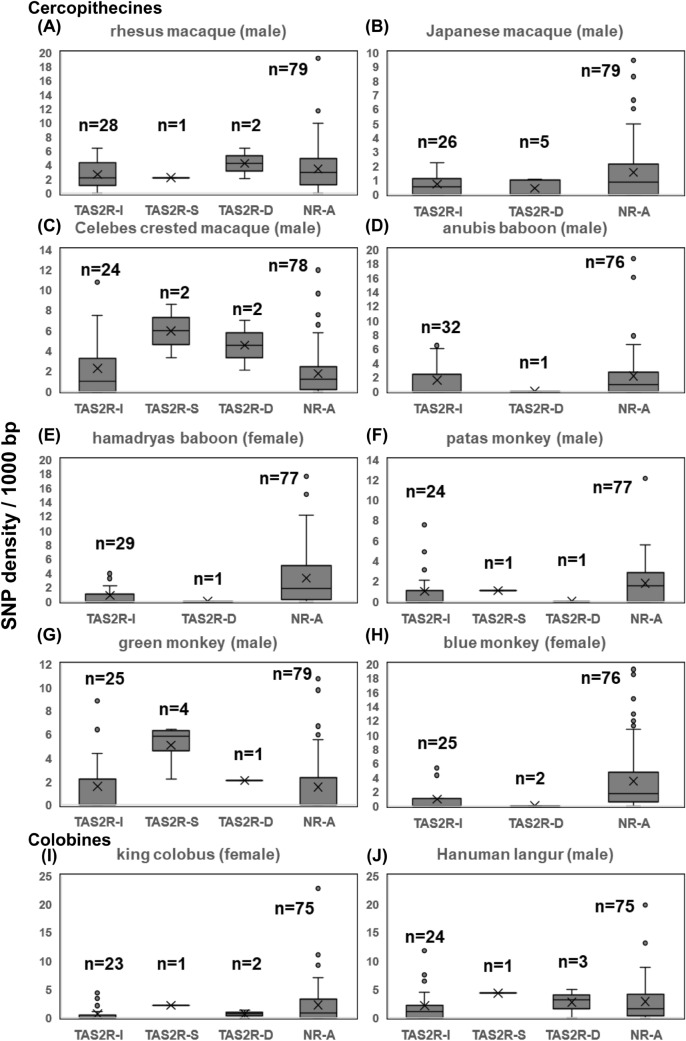
The range of final SNP density values of intact (TAS2R-I), segregating pseudogene (TAS2R-S), and disrupted (TAS2R-D) ancestral-cercopithecid and single-locus *TAS2R* genes and that of autosomal neutral references (NR-A) in each cercopithecid study species, depicted by boxplots. **A**–**H** are cercopithecine species: **A** rhesus macaque, **B** Japanese macaque, **C** Celebes crested macaque, **D** anubis baboon, **E** hamadryas baboon, **F** patas monkey, **G** green monkey, and **H** blue monkey. **I** and **J** are colobine species: **I** king colobus and **J** Hanuman langur. The medians are indicated as the horizontal line in the box. The means are indicated as the symbol “X.” The upper whisker value represents the largest within 1.5 times interquartile range above the third quartile, and the lower whisker value represents the smallest within 1.5 times interquartile range below the first quartile. The outliers are indicated by dots. The median was included in the calculation to determine quartile values. The number of data points are indicated with *n*

The final per-site sequencing depth values of the *TAS2R* genes and the neutral references are listed in Tables S6-1 and S6-2, respectively. The per-site sequencing depth data of the single-locus and ancestral-cercopithecid *TAS2R* genes averaged in each species were summarized in Table 3. The range of the final sequencing depth of *TAS2R* genes in the ancestral-cercopithecid *TAS2R* gene set, that of the NR-As and that of the X-chromosomal neutral references (NR-Xs) in each cercopithecid species are indicated with boxplots (Fig. 2). The final SNP density values of intact, disrupted, and segregating pseudogene categories (see the section “Classification of *TAS2R* sequences into intact and disrupted types” and Table S7) of ancestral-cercopithecid *TAS2R* genes in each species are listed in Tables S8-1–10. Among the data, those of the single-locus *TAS2R* genes are box-plotted with SNP density of the single-locus NR-As in each species (Fig. 3).

### Classification of *TAS2R* sequences into intact and disrupted types

We used the EMBOSS getorf to find ORF (Rice et al. 2000). We regarded any *TAS2R* sequence with maximum ORF < 750 bp (encoding < 250 amino acids long) as disrupted because such a sequence is unlikely to encode a functional structure (Hayakawa et al. 2014). We also examined if a TAS2R sequence was capable of forming the 7-TM domain, a hallmark of GPCRs, by using DeepTMHMM (Hallgren et al. 2022). We regarded any sequence without proper 7-TM domain as disrupted. Thus, we regarded only *TAS2R* sequences with an ORF of at least 750 bp and capable of forming a 7-TM domain as intact.

ORF and 7-TM domain cannot be predicted appropriately for the *TAS2R* sequences containing IUPAC degenerate nucleotide codes assigned to SNP sites among which haplotype phases were not determinable. In such cases, for a pair of H1 and H2 sequences, we replaced an IUPAC degenerate code (e.g., R for A and G) in H1 with a nucleotide code chosen in alphabetical order (A in this example) (H1-1) and that in H2 with another nucleotide (G) (H2-1). We also made a replacement in the opposite combination, G in H1 (H1-2) and A in H2 (H2-2), in this example because ORF and 7-TM domain can be disturbed by one amino acid site irrespective of haplotype phases. The combination of nucleotides between IUPAC degenerate code sites increases exponentially as the number of such sites increases. Rather than testing all the possible combinations exhaustively, we tested the two pairs (H1-1/H2-1 and H1-2/H2-2) throughout all degenerate sites in the same way (for example, suppose there are two degenerate code sites, R (A and G) and Y (C and T), one combination between two sites is in alphabetical order, i.e., A and C in H1 allele for the R and Y sites, respectively, and G and T in H2 allele. Another combination was in the opposite order, i.e., G and T in H1 allele and A and C in H2 allele).

Table S7 summarizes the ORF length and the predicted number of TM domains of H1-1, H2-1, H1-2, and H2-2 sequences for the *TAS2R* genes that we considered to exist based on sequencing depth in the mapping procedures (Table S6-1). If there was no site with IUPAC degenerate nucleotide codes and if H1 and H2 sequences were identical, ORF length and the predicted number of TM domains were given only to the H1-1 column. If there was no site with IUPAC degenerate nucleotide codes and if H1 and H2 sequences were different, ORF length and the predicted number of TM domains were given only to the H1-1 and H2-1 columns.

If all sequences of a gene were intact, we regarded the gene as intact (Tables S7-1–10). If all sequences were disrupted, we regarded the gene as disrupted (Tables S7-1–10). Regarding the single-locus genes, if both intact and disrupted forms were found, we regarded the gene as heterozygous with intact and disrupted alleles and we designated such *TAS2R* gene as a segregating pseudogene (Tables S7-1–10). Regarding categories 1 and 3 duplicated/multiplicated *TAS2R* genes and category 4 genes with high SNP density but not with high sequencing depth (Table 2). “Intact” and “disrupted” mean that the congregated sequence of potentially paralogous and allelic origins is comprised of only intact and disrupted sequences, respectively. On the other hand, “intact and disrupted” means that the congregated sequence is composed of intact and disrupted sequences but it remains uncertain whether intact and disrupted sequences are alleles of a locus or paralogous genes.

### Reconstruction of phylogenetic trees

**Fig. 4:**
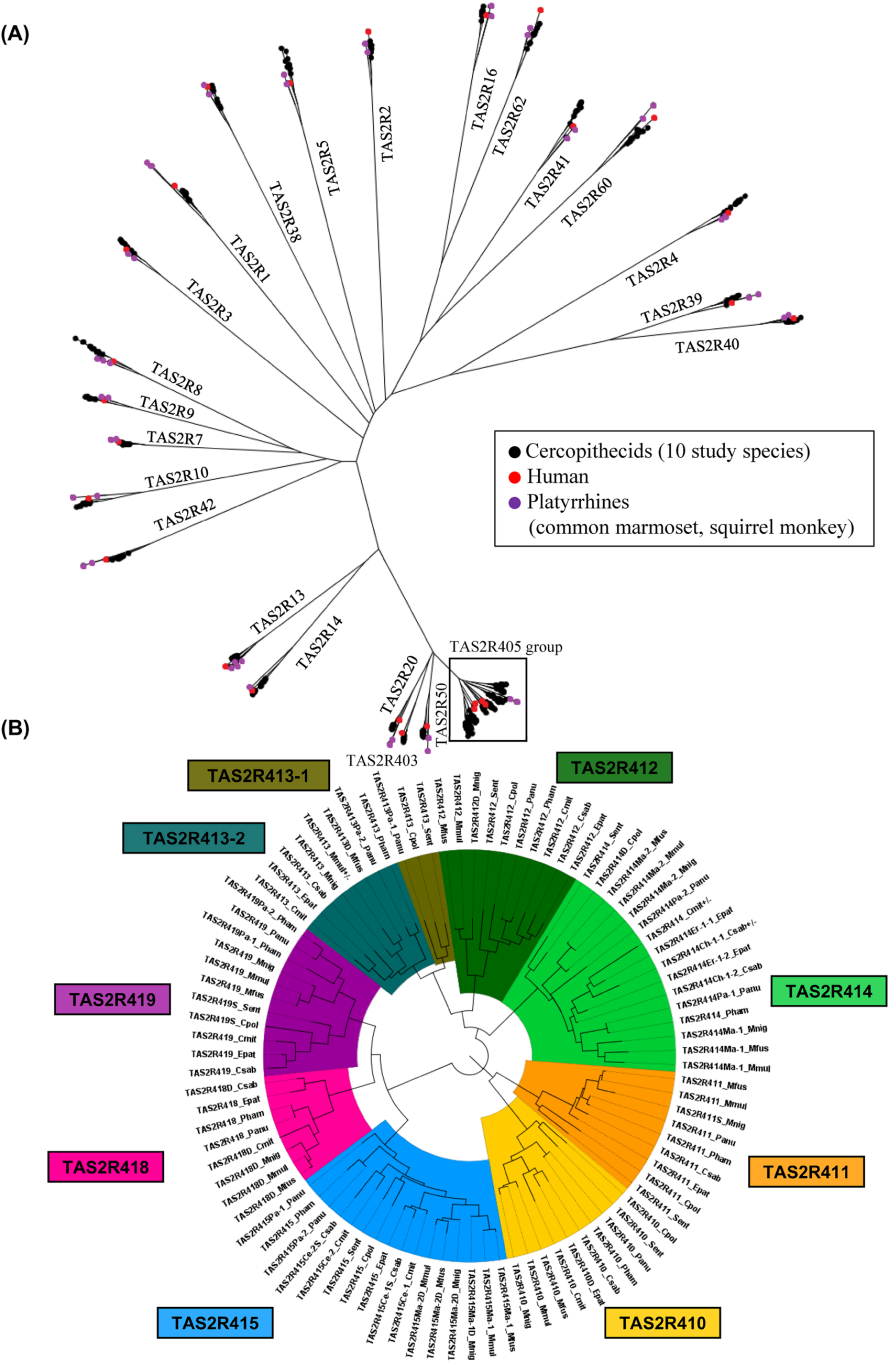
Phylogenetic trees reconstructed for the ancestral-cercopithecid *TAS2R* gene set. Regarding the single-locus *TAS2R* genes, only one of the two allele sequences (H1) was used to represent each gene. Regarding categories 1 and 3 duplicated/multiplicated *TAS2R* genes and category 4 genes high SNP density but not with high sequencing depth (Table 2), the congregated sequence of each gene group was used. **A** A phylogenetic tree covering entire ancestral-cercopithecid *TAS2R* gene set. Cercopithecid genes are symbolized with black dots. As outgroups, known orthologous gene sequences (Hayakawa et al. 2014) available from human (red dots) and platyrrhines (common marmoset and squirrel monkey) (purple dots) are used. **B** An expanded view of the “*TAS2R405*” group phylogenetic tree. The nine orthologous genes (*TAS2R410*, *TAS2R411*, *TAS2R412*, *TAS2R413-1*, *TAS2R413-2*, *TAS2R414*, *TAS2R415*, *TAS2R418*, and *TAS2R419*) are differently colored for easier distinction. Note that human and platyrrhine out-group sequences are not included in (**B**) because the nine genes occurred at the common ancestor of cercopithecids after its separation from the hominoid ancestor (and thus after separation from platyrrhine ancestor), and there is no corresponding human and platyrrhine sequences to each of the nine genes. A sequence name consists of the gene name followed by the species name abbreviation (e.g., *TAS2R410* Mmul; see Table 1 for abbreviation). The disrupted genes are suffixed with “D” after the gene name. Segregating pseudogenes are suffixed with “S” after the gene name. The congregated sequences for which both intact and disrupted sequences were inferred (Table S7) are labeled ± after the sequence names

We aligned nucleotide sequences using MUSCLE (Edgar 2004). We estimated the number of nucleotide substitutions per nucleotide site between two sequences using the Tamura-Nei model (Tamura and Nei 1993) with gap sites excluded in pairwise fashion. We reconstructed phylogenetic trees using the neighbor-joining method (Saitou and Nei 1987) and evaluated the reliability of the trees by bootstrap resampling with 1000 replications (Felsenstein 1985) (Figs. 4 and S4). These procedures were conducted under the platform of MEGA11 (Tamura et al. 2021).

### Counting gene birth and gene death events

**Fig. 5:**
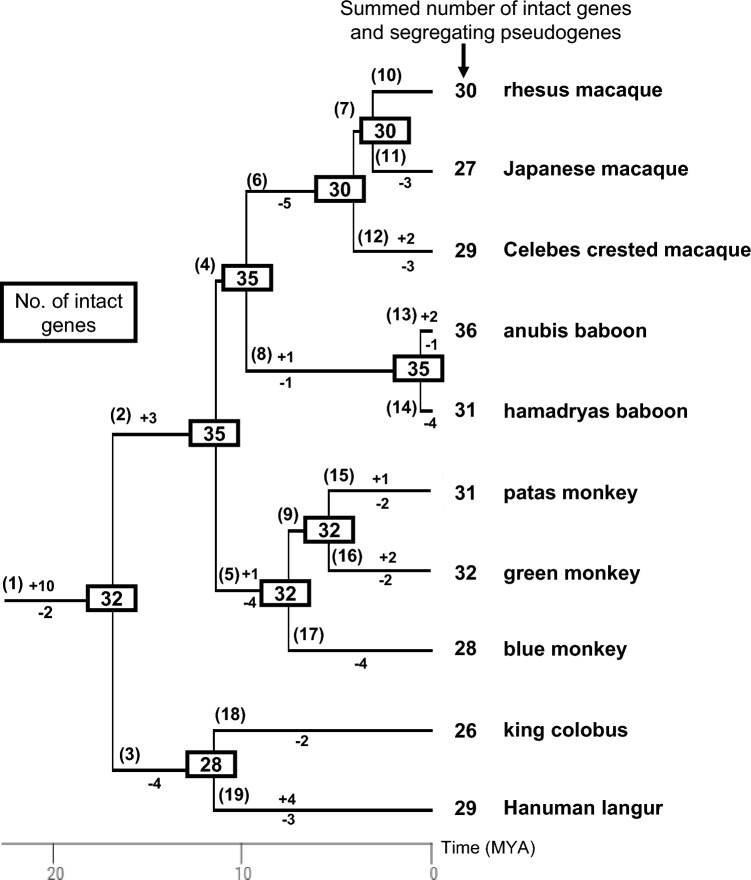
Evolutionary birth and death process of ancestral-cercopithecid *TAS2R* gene set. The total numbers of intact genes and segregating pseudogenes of the study species are indicated beside their common names. The numbers of intact genes inferred to common ancestors are boxed. The numbers of gene births and gene deaths at every branch are indicated with plus (+) and minus (−) codes, respectively. The numbers in parentheses represent the branch IDs shared with Table S9. The phylogenetic relationship and divergence times were given by using TimeTree (Kumar et al. 2017) and modified based on literature (Li et al. 2009; Perelman et al. 2011)

Based on Fig. S4 (1) ~ (30), the evolutionary timing of gene “births” (gain by gene duplication) and gene “deaths” (disruption or loss) was inferred (Fig. 5 and Table S9). The births were considered only for the genes listed in Table 2 as categories 1, 2, and 3 (*TAS2R10*, *TAS2R14*, *TAS2R50*, *TAS2R60*, *TAS2R413*, *TAS2R414*, *TAS2R415*, and *TAS2R419*) for which gene duplications/multiplications were inferred based on the sequencing depth and the SNP density. Among the other *TAS2R* genes, there were genes (*TAS2R1*, *TAS2R2*, *TAS2R4*, *TAS2R8*, *TAS2R9*, *TAS2R13*, *TAS2R38*, *TAS2R42*, *TAS2R403*, *TAS2R412*, and *418* in Fig. S4) for which the tree topology was not congruent with the phylogenetic relationship among species (Fig. 1). Because incongruency with the species tree could arise due to various causes such as incomplete lineage sorting or low statistical support for the tree topology, we did not assume “hidden” gene duplications after which one of paralogous genes were lost. The timing of gene duplication was inferred based on the maximum parsimony principle so that the sum of the number of gene birth and death events was the minimum under the assumption of frequent occurrence of homogenization between duplicated genes by gene conversion (Hiwatashi et al. 2011). For example, in the *TAS2R415* gene tree [Fig. S4 (28)], we assumed a gene duplication once at the common ancestor of cercopithecines (Table S9) and subsequent gene conversions at the common ancestor of *Macaca* species, at that of *Papio* species and at that of the tribe Cercopithecini species (patas, green, and blue monkeys) rather than gene duplications at each of these three lineages.

**Fig. 6:**
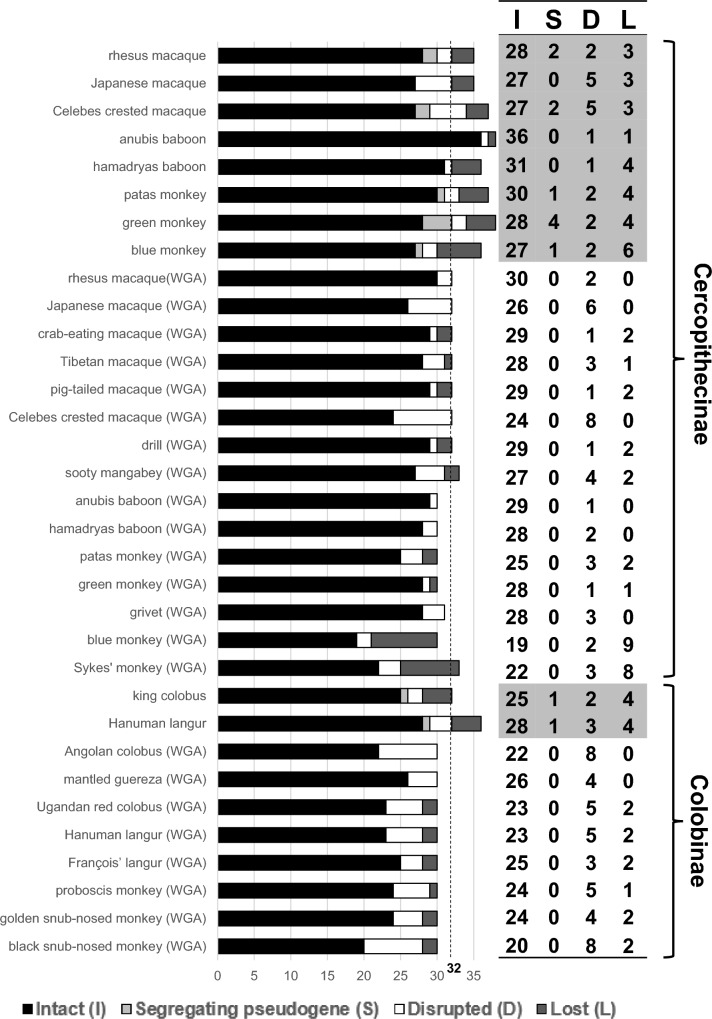
The numbers of intact, segregating pseudogene, disrupted, and lost *TAS2R* genes in cercopithecid primates relative to the 32 ancestral-cercopithecid *TAS2R* genes. The numbers in ten study species are highlighted with gray shadow. The number of intact, disrupted, and absent (lost) *TAS2R* genes searched from the public WGA databases are also shown

At each tree branch of Fig. 5, the number of intact genes gained or lost/disrupted is indicated with plus (+) or minus (−) symbol, respectively. Segregating-pseudogenizations of single-locus genes were not regarded as gene deaths and were not included as losses. Thus, in Fig. 5 the number of intact genes at tree nodes could include segregating pseudogenes. Gene duplication/multiplication events (categories 1, 2, and 3) were each counted as +1. Category 4 genes were not counted as gains. Disruption (see Table S7) or loss of single-locus genes, category 2 genes, or category 4 genes were each counted as −1. If category 1 or 3 genes had only disrupted sequences (see Table S7), disruptions were counted as −2. If category 1 or 3 genes had both intact and disrupted sequences (see Table S7), disruptions were counted as −1. If category 4 genes had both intact and disrupted sequences (see Table S7), these were not regarded as gene deaths and were not counted as losses. Thus, these were included in the total numbers of intact genes and segregating pseudogenes of the study species, indicated beside their common names in Fig. 5 and in segregating pseudogenes in Fig. 6.

## Results

### Inference of gene duplications and disruptions for ancestral-cercopithecid *TAS2R* gene set

Using the probes designed for the *TAS2R* genes that were inferred to be intact at the last common ancestor of cercopithecid primates (designated as “ancestral-cercopithecid *TAS2R* gene set”; see Materials and Methods: Table S2-1) and the probes designed for neutral references (Table S2-2), we conducted the targeted capture for ten cercopithecid (eight cercopithecine and two colobine) species (Table 1, Fig. 1). Using up to five rounds of mapping and assemblage of NGS short reads, we retrieved nucleotide sequences of *TAS2R* genes and neutral references for the ten study species [Supplementary Data 1 (ancestral-cercopithecid *TAS2R* genes) and 2 (neutral references)].

Gene duplications/multiplications were inferred for eight genes (*TAS2R10*, *TAS2R14*, *TAS2R50*, *TAS2R60*, *TAS2R413*, *TAS2R414*, *TAS2R415*, and *TAS2R419*) as category 1, 2, or 3 (Table 2) based on the evaluation of sequencing depth and SNP density (see Materials and Methods). However, there were genes for which SNP density was high but sequencing depth was not, after the final-round of mapping (category 4 in Table 2). With the current dataset, we waived the decision on whether these genes were duplicated/multiplicated or single locus.

We classified the single-locus *TAS2R* genes into not only intact and disrupted genes but also segregating pseudogenes (genes heterozygous with intact and disrupted alleles) in each species (Tables S7-1–10). The latter is not reliably retrievable from raw NGS short-read data in many public WGA databases due to their low sequencing depth. The congregated sequences of categories 1, 3, and 4 genes were also classified into intact, disrupted, or both intact and disrupted with paralogous and allelic origins mixed (Tables S7-1–10).

Regarding *TAS2R* genes for which probes were prepared (Table S2-1), there was no gene for which the intact sequences were not retrieved from any of the ten study species (Table S7). Regarding *TAS2R* genes for which probes were not prepared (Table S3), all genes but *TAS2R418* were either absent or disrupted (Tables S6, S7). *TAS2R418* was found to be intact in anubis baboon (Table S7-4), hamadryas baboon (Table S7-5), and patas monkey (Table S7-6) samples, evidencing that this gene was intact at the last common ancestor of cercopithecid primates as Hayakawa et al. (2014) assumed. All nonprobed *TAS2R* genes except *TAS2R418* were absent or disrupted in all of cercopithecid WGA databases (Table S1-2) currently available. These results support that all probed *TAS2R* genes and *TAS2R418*, but not other *TAS2R* genes, compose the ancestral-cercopithecid *TAS2R* gene set.

### Sequencing depth and SNP density

The per-site sequencing depth values of the *TAS2R* genes and the neutral references are listed in Tables S6-1 and S6-2, respectively. Sequencing depth of *TAS2R* genes (all autosomal) in the ancestral-cercopithecid *TAS2R* gene set was largely similar to that of the autosomal neutral references (NR-As) (assumed to be single-locus; see Materials and Methods) if excluding the upper outliers of *TAS2R* genes, which we regarded as duplicated or multiplicated (see Materials and Methods) (Fig. 2). The sequencing depth of the X-chromosomal neutral references (NR-Xs) was roughly half of the NR-As in the samples from male individuals (rhesus macaque, Japanese macaque, Celebes crested macaque, anubis baboon, green monkey, patas monkey, and Hanuman langur) whereas that of the NR-X was similar with that of the NR-As in the samples from female individuals (blue monkey, hamadryas baboon, and king colobus) (Fig. 2). These observations supports the supposition that sequencing depth can reflect the gene ploidy difference in our samples.

The average per-site sequencing depth values of the single-locus and ancestral-cercopithecid *TAS2R* genes in each species (353 in the patas monkey sample, 1224–1696 in the others, and 1286 on average) were overall much higher than those available in the public WGA database (29–83, with 54 on average) (Table 3). Among SNP density values of NR-As and the single-locus and ancestral-cercopithecid *TAS2R* genes, SNP density was generally highest in NR-As in each species, which is consistent with expectation given the difference in functional constraint (Fig. 3, Tables S8-1–10).

**Table 3:** The per-site sequence depth of single-locus and ancestral-cercopithecid *TAS2R* genes retrieved by targeted capture (TC) in this study and of public WGA databases of study species

Subfamily	Genus	Species	Common name	TC	WGA
Cercopithecinae	*Macaca*	*Mulatta*	Rhesus macaque	1480	66
		*Fuscata*	Japanese macaque	1243	42
		*Nigra*	Celebes crested macaque	1224	31
	*Papio*	*Anubis*	Anubis baboon	1372	60
		*Hamadryas*	Hamadryas baboon	1328	51
	*Erythrocebus*	*Patas*	Patas monkey	353	52
	*Chlorocebus*	*Sabaeus*	Green monkey	1398	75
	*Cercopithecus*	*Mitis*	Blue monkey	1696	83
Colobinae	*Colobus*	*Polykomos*	King colobus	1436	NA*
	*Semnopiethecus*	*Entellus*	Hanuman langur	1333	29
			Average	1286.3	54.3

*Data not available

### Evolutionary birth and death process of *TAS2R* genes in cercopithecid primates

Figure 4A shows a phylogenetic tree reconstructed for entire ancestral-cercopithecid *TAS2R* gene set and Fig. 4B provides an expanded view of the “*TAS2R405* group” while Fig. S4 provides subtrees for each gene group. We observed clustering of the *TAS2R* genes retrieved in this study with their known orthologous outgroups, supporting their orthology identification (Fig. 4A). Based on the subtrees [Fig. S4 (1) ~ (30)], evolutionary timing of gene “birth” (gain by gene duplication) and gene “death” (disruption or loss) was inferred. Among the *TAS2R405* group genes (Fig. 4), *TAS2R416* and *TAS2R417* were previously considered to have arisen in the common ancestor of cercopithecids in Hayakawa et al. (2014), where WGA databases of two macaque and one baboon species were studied. Including more diverse cercopithecid species in this targeted-capture study, we found that these two genes arose within the *TAS2R415* at the common ancestor of cercopithecines, and we renamed *TAS2R416* as *TAS2R415-1* and *TAS2R417* as *TAS2R415-2* [Fig. S4 (28)]. On the other hand, we found that *TAS2R10* and *TAS2R413* were duplicated to *TAS2R10-1* and *TAS2R10-2* [Fig. S4 (9)] and *TAS2R413-1* and *TAS2R413-2* [Fig. S4 (26) and Fig. 4B], respectively, at the common ancestor of cercopithecids. These duplications were not detected by the previous WGA-based study on the three cercopithecine species (Hayakawa et al. 2014).

Figure 5 depicts the birth and death process of the ancestral-cercopithecid *TAS2R* gene set. Table S9 summarizes the genes gained by gene duplications and the genes disrupted or lost at every branch in Fig. 5. Gene “birth” or “death” events occurred at almost every branch (Fig. 5), making the composition of intact genes variable among species. At the common ancestor of cercopithecids (Branch 1 in Fig. 5), ten births were inferred (Table S9). Eight of the ten were from the *TAS2R405* group, including *TAS2R413-1* and *TAS2R413-2* as noted above. Another was from *TAS2R10*, giving rise of *TAS2R10-1* and *TAS2R10-2*, as also noted above. The last one was according to Hayakawa et al. (2014) and was from *TAS2R409*, giving rise of *TAS2R403* and *TAS2R404* with the latter being lost in the same branch (Table S9). Thus, the number of births in branch 1 happened to be the same with the inference by Hayakawa et al. (2014). At this branch, another death occurred (*TAS2R15*), according to Hayakawa et al. (2014). The number of intact genes at the common ancestor of cercopithecids were inferred to be 32, as Hayakawa et al. (2014) inferred.

At the common ancestor of cercopithecines (branch 2 in Fig. 5), three gene births from *TAS2R50*, *TAS2R414*, and *TAS2R415* were inferred, whereas at the common ancestor of colobines (branch 3 in Fig. 5), four gene deaths [one disruption (*TAS2R403*) and three losses (*TAS2R10-2*, *TAS2R413-2*, and *TAS2R418*)] were inferred (Table S9). Overall, the total number of intact genes and segregating pseudogenes were smaller in colobines (26–29) than in cercopithecids (27–36) (Fig. 5).

In Fig. 6, the numbers of intact, segregating pseudogene, disrupted, and lost *TAS2R* genes relative to the 32 ancestral-cercopithecid *TAS2R* genes are band-graphed for cercopithecid species examined in this study. The number of intact genes were 27–36 in cercopithecids and 25–28 in colobines (Fig. 6). Figure 6 also indicates the number of intact, disrupted, and absent (lost) *TAS2R* genes searched from the public WGA databases. The trend of less intact *TAS2R* genes in colobines than in cercopithecines was also observed in the public WGA databases: 20–26 in colobines and 19–29 in cercopithecines. Relative to the 32 intact genes at the cercopithecid common ancestor, the anubis baboon increased to 36 with no segregating pseudogenes, and the green monkey retained 32, including segregating pseudogenes. The others have decreased the total number of intact genes and segregating pseudogenes in both cercopithecines and colobines. The decrease appeared to be more evident in colobines.

### *TAS2R* gene retrieval compared with WGA-database search

The “intact” genes of our study represent only genes homozygous with intact alleles whereas those of WGA databases could not only represent them but also include intact alleles from segregating pseudogenes. Nevertheless, the number of intact genes were more in our targeted-capture-based study (25–36) than in WGA-based search (19–29) (Fig. 6).

A gene-by-gene comparison of intact/disrupted/segregating pseudogene/lost (absence) statuses of *TAS2R* genes between WGA and targeted-capture is summarized in Table S10. There were ten genes that were disrupted in the WGA databases but were intact or duplicated with intact and disrupted genes in our targeted capture [one in Japanese macaque (*TAS2R1*), four in Celebes crested macaque (*TAS2R8*, *TAS2R38*, *TAS2R42*, *TAS2R419*), one in hamadryas baboon (*TAS2R3*), one in patas monkey (*TAS2R413*), three in Hanuman langur (*TAS2R14*, *TAS2R42*, and *TAS2R60*)]. There were nine genes that were absent in the WGA database but were intact in our targeted capture [two in patas monkey (*TAS2R414* and *TAS2R418*), seven in blue monkey (*TAS2R1*, *TAS2R3*, *TAS2R4*, *TAS2R7*, *TAS2R8*, *TAS2R9*, and *TAS2R10*)]. There was one gene that was absent in WGA database but was multiplicated with intact and disrupted genes in our targeted capture [green monkey (*TAS2R414*)]. There were five genes which were intact in the WGA databases and were segregating pseudogenes in our targeted capture [two in rhesus macaque (*TAS2R5* and *TAS2R413*), one in green monkey (*TAS2R60*), one in blue monkey (*TAS2R414*), and one in Hanuman langur (*TAS2R419*)], whereas there was no gene which was intact in the WGA databases but was lost or disrupted in our targeted capture. These results indicate that our targeted capture retrieved more *TAS2R* genes and characterized their intact/disrupted and duplication statuses better than WGA databases.

## Discussion

In this study we employed the targeted capture to specifically probe the *TAS2R* genes and the autosomal and X-chromosomal neutral reference regions from genomic DNA samples of ten Cercopithecidae primates including eight omnivorous Cercopithecinae and two folivorous Colobinae species. The targeted capture was followed by short-read and high-depth massive-parallel sequencing. We confirmed that the sequencing depth well reflected the ploidy difference of X-chromosomal neutral references between males and females relative to autosomal regions, validating the utility of sequencing depth as an indicator of gene number differences in each sample. The sequencing depth achieved by the targeted capture was roughly 20 times higher than that of publicly available cercopithecid WGA databases, consolidating reliability of our sequencing results. Consistently, our targeted capture retrieved more intact *TAS2R* genes and characterized their intact/disrupted and duplication statuses better than WGA databases.

We designed probes for all *TAS2R* genes that were inferred to be intact at the common ancestor of cercopithecids (designated as “ancestral-cercopithecid *TAS2R* gene set”). By using this phylogeny-based approach, we aimed to depict the evolutionary trajectory of the gene set, i.e., which genes increased their numbers by gene duplications (gene birth) and which genes were disrupted or lost (gene death). Disrupted genes (pseudogenes) already present in the common ancestor of cercopithecid primates were not considered in this study. This was because they should remain inactive thereafter and be irrelevant to the evolution and diversification of taste receptor function in cercopithecid primates. Inclusion of such pseudogenes at the common ancestor of study species, which previous WGA-based studies have done (Li and Zhang 2014; Liu et al. 2016; Shi and Zhang 2006), could ambiguate the significance of the interspecies comparison of pseudogene numbers.

We were surprised to find intact *TAS2R418* genes in our samples (anubis baboon, hamadryas baboon, and patas monkey). We did not include the *TAS2R418* gene in our probe set because the *TAS2R418* gene was previously found disrupted in the three cercopithecine and the two colobine WGA databases we used for designing probes (Hayakawa et al. 2014). It was our fortune that the gene was captured by other gene probes, possibly the *TAS2R419* probe designed from rhesus macaque, for which the sequence difference was only 7–8% different from *TASR418*, well within the range of ~10% mismatch allowed between baits and targets. This supports Hayakawa et al’s (2014) hypothesis that it was intact (or at least a segregating pseudogene) in the last common ancestor of Cercopithicidae.

Li and Zhang (2014) reported that the number of *TAS2R* genes in vertebrates was positively correlated with the fraction of dietary plants by studying 54 WGA databases. This led to a general prediction that herbivores may need more *TAS2R* genes to recognize a larger number of bitter compounds than carnivores and omnivores to select the type of plants they eat (Li and Zhang 2014). This appears to be consistent with the large number of gene birth at the common ancestor of cercopithecids. As Hayakawa et al. (2014) and Toda et al. (2021) discussed, progressive increase in body size of ancestral cercopithecid primates during the Oligocene epoch may have been associated with a change of protein source from insects to leaves although they were not committed folivores (Kay et al. 1997; Williams et al. 2010).

While cercopithecid primates have maintained folivory as a part of their omnivorous diets, colobine primates are specialized for folivory as their main diet. However, opposed to the herbivory prediction, the number of intact *TAS2R* genes was overall smaller in folivorous colobines than in omnivorous cercopithecines. Glendinning (1994) showed that herbivorous mammals, especially browsers, which commonly encounter bitter and potentially poisonous foods, had a high bitter threshold (i.e., low bitter sensitivity) and high tolerance to ingest toxic compounds. This was explained as herbivores may have evolved detoxification mechanisms, such as the foregut fermentation by microbes in ruminants, to tolerate toxic compounds (Freeland and Janzen 1974). Colobine monkeys have also independently evolved a foregut-fermentation digestive system and use symbiont microorganisms to detoxify plant compounds (Bauchop and Martucci 1968). The foregut bacteria are digested by the specialized lysozyme expressed in the stomach to recover nutrients (Messier and Stewart 1997). The bacterial RNA molecules are degraded in the small intestine by the specialized RNASE1B to recycle nitrogen efficiently (Zhang et al. 2002). A fewer number of intact *TAS2R* genes in colobines than in cercopithecines appears to be congruent with the Glendinning’s (1994) observation. Previous research (Purba et al. 2017) identified four nonsynonymous nucleotide substitutions in the colobine *TAS2R38* gene that were responsible for the decreased sensitivity of the TAS2R38 to phenylthiocarbamide (PTC). Furthermore, compared with macaque monkeys, colobines have lower sensitivities to PTC, which can be recognized by TAS2R38 in behavioral and in vitro functional analyses (Purba et al. 2020). These findings support the hypothesis that colobines have developed a tolerance to bitterness as an adaptation to their leaf-eating dietary specialization. Overall, our results show that the evolutionary change of number of intact *TAS2R* genes is a complex process including evolutionary acquisition of detoxication ability and refute a simple general prediction that herbivores need more *TAS2R* genes.

Our study has revealed far greater variation and complexity in the composition of *TAS2R* genes among primates. Indeed, we found that birth or death events occurred at almost every phylogenetic-tree branch in the species we studied. Such a discovery welcomes additional research to understand this dynamic evolutionary process. Bitter compounds have been identified for various *TAS2R*s by heterologous expression of their genes in cultured cells and by behavioral tests (Itoigawa et al. 2021; Meyerhof et al. 2010; Purba et al. 2020; Tsutsui et al. 2016). Further clarification of the *TAS2R* gene repertoire, ligands of the receptors, and bitterness perception compared between cercopithecines and colobines and among species aligned with their distinct dietary strategies will further elucidate the evolution of bitter taste receptors in relation to feeding ecology.

### Supplementary Information

Below is the link to the electronic supplementary material.

Supplementary data1 (TXT 683 KB)

Supplementary data 2 (TXT 4979 KB)

Supplementary figures 1–4 (PDF 739 KB)

Supplementary table 1 (XLSX 17 KB)

Supplementary table 2 (XLSX 28 KB)

Supplementary table 3 (XLSX 12 KB)

Supplementary table 4 (XLSX 28 KB)

Supplementary table 5 (XLSX 47 KB)

Supplementary table 6 (XLSX 31 KB)

Supplementary table 7 (XLSX 35 KB)

Supplementary table 8 (XLSX 31 KB)

Supplementary table 9 (XLSX 12 KB)

Supplementary table 10 (XLSX 12 KB)

Supplementary table 11 (XLSX 11 KB)

## Data Availability

This is not applicable.
