# Supplementary material for: Reduction of bitter taste receptor gene family in folivorous colobine primates relative to omnivorous cercopithecine primates
Source: Primates. 2024 Apr 11;65(4):311–31. doi: 10.1007/s10329-024-01124-w (PMC11219393; doi:10.1007/s10329-024-01124-w)
Supplement: Supplementary file 3 — Supplementary figures 1–4 (PDF 739 KB) [file 10329_2024_1124_MOESM3_ESM.pdf]

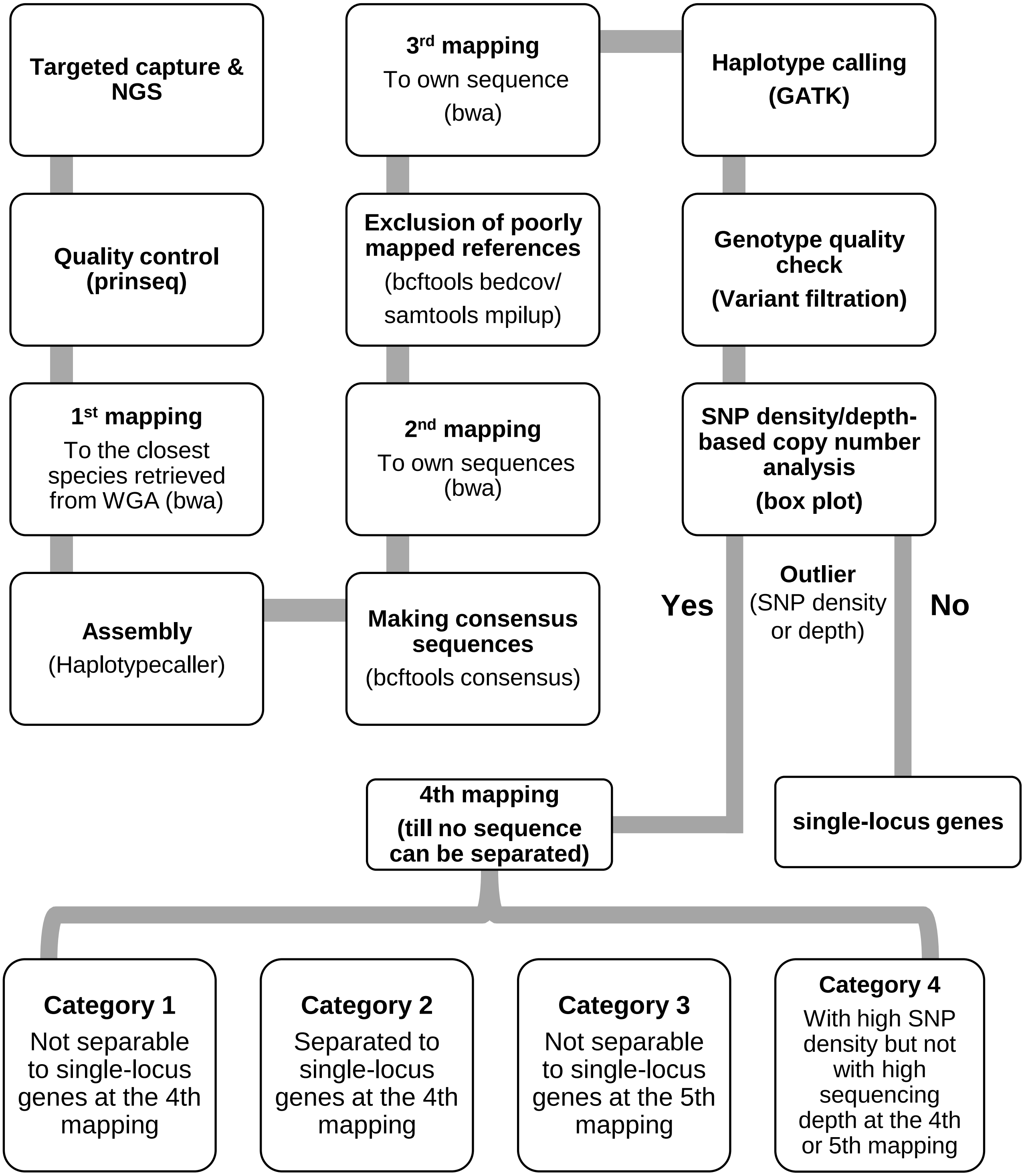


Hou et al. Fig. S1

**Fig. S1.** Flowchart of analytical procedure.

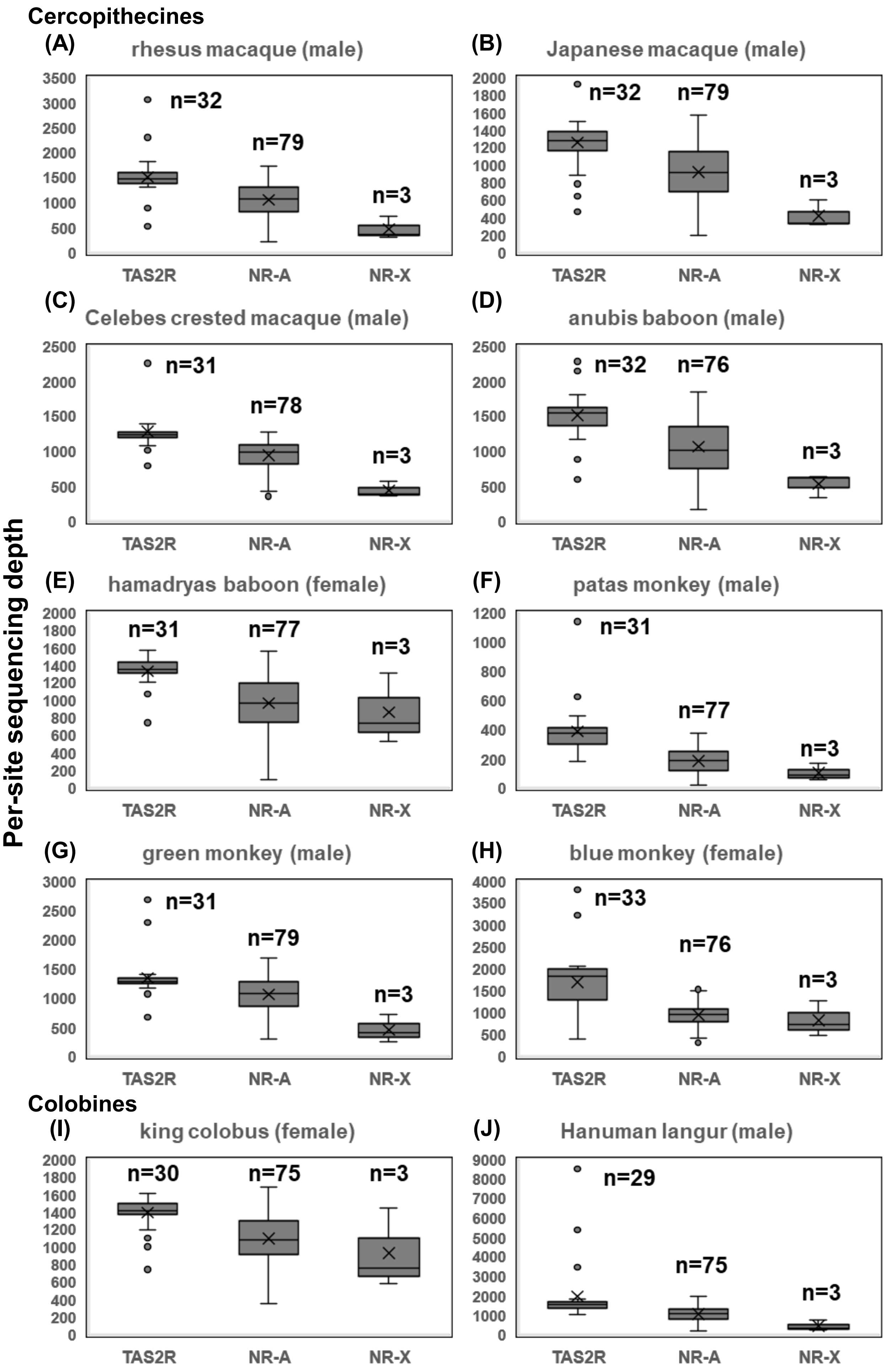


Hou et al. Fig. S2

**Fig. S2.** The range of per-site sequencing depth at the third-round mapping and the subsequent trimming of the ancestral-cercopithecid *TAS2R* genes, that of autosomal neutral references (NR-A) and that of the X-chromosomal neutral references (NR-Xs) in each cercopithecid study species depicted by boxplots. (A)~(H) are cercopithecine species: (A) rhesus macaque, (B) Japanese macaque, (C) Celebes crested macaque, (D) anubis baboon, (E) hamadryas baboon, (F) patas monkey, (G) green monkey, and (H) blue monkey. (I) and (J) are colobine species: (I) king colobus, (J) Hanuman langur. The medians are indicated as the horizontal line in the box. The means are indicated as the symbol 'X'. The upper whisker value represents the largest within 1.5 times interquartile range above the third quartile, and the lower whisker value represents the smallest within 1.5 times interquartile range below the first quartile. Outliers are indicated by dots. The median was included in the calculation to determine quartile values. The number of data points are indicated with *n*.

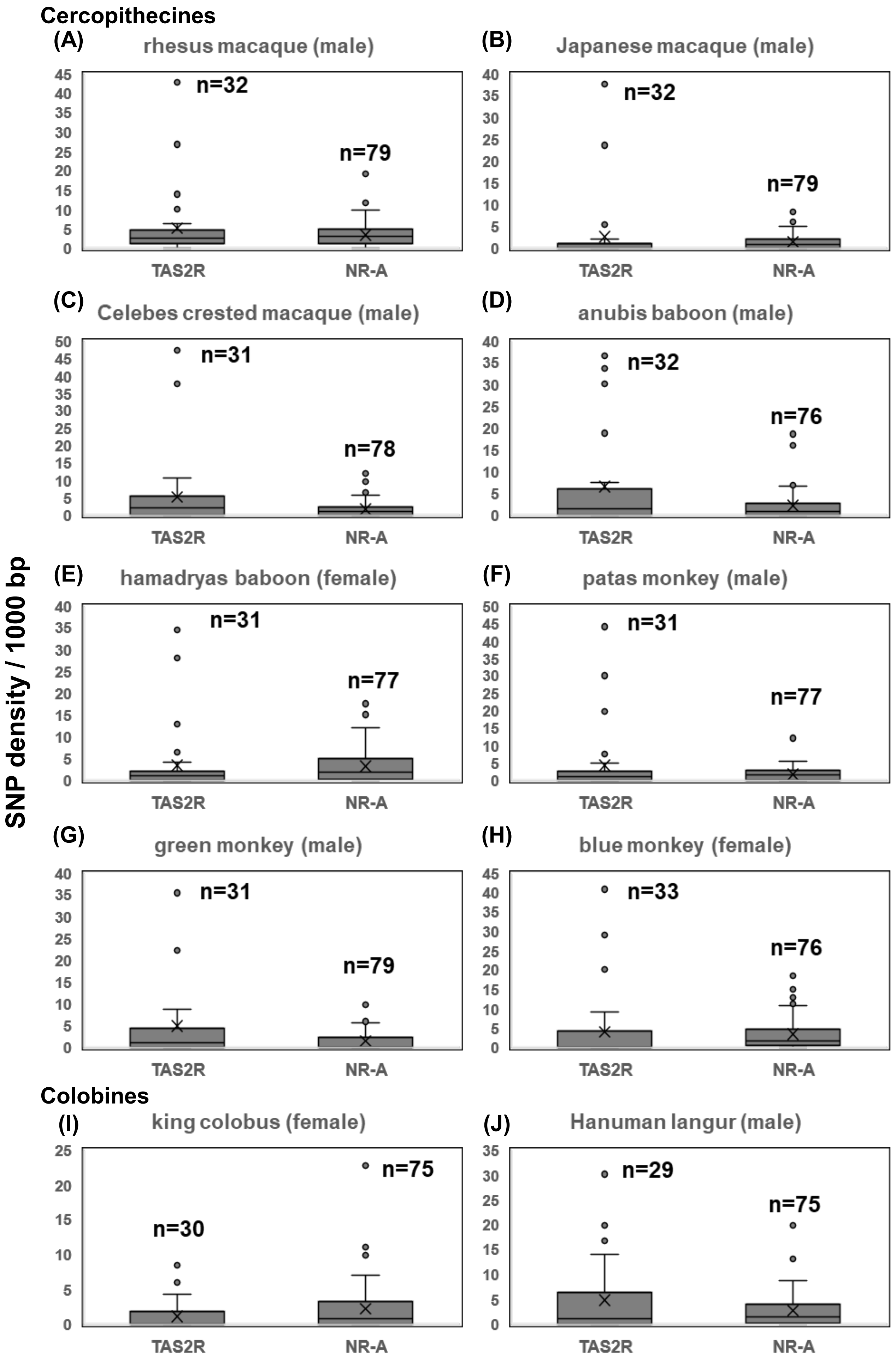


Hou et al. Fig. S3

**Fig. S3.** The range of SNP density values at the third-round mapping and the subsequent trimming of the ancestral-cercopithecid *TAS2R* genes and that of autosomal neutral references (NR-A) in each cercopithecid study species depicted by boxplots. (A)~(H) are cercopithecine species: (A) rhesus macaque, (B) Japanese macaque, (C) Celebes crested macaque, (D) anubis baboon, (E) hamadryas baboon, (F) patas monkey, (G) green monkey, and (H) blue monkey. (I) and (J) are colobine species: (I) king colobus, (J) Hanuman langur. The medians are indicated as the horizontal line in the box. The means are indicated as the symbol 'X'. The upper whisker value represents the largest within 1.5 times interquartile range above the third quartile, and the lower whisker value represents the smallest within 1.5 times interquartile range below the first quartile. Outliers are indicated by dots. The median was included in the calculation to determine quartile values. The number of data points are indicated with *n*.

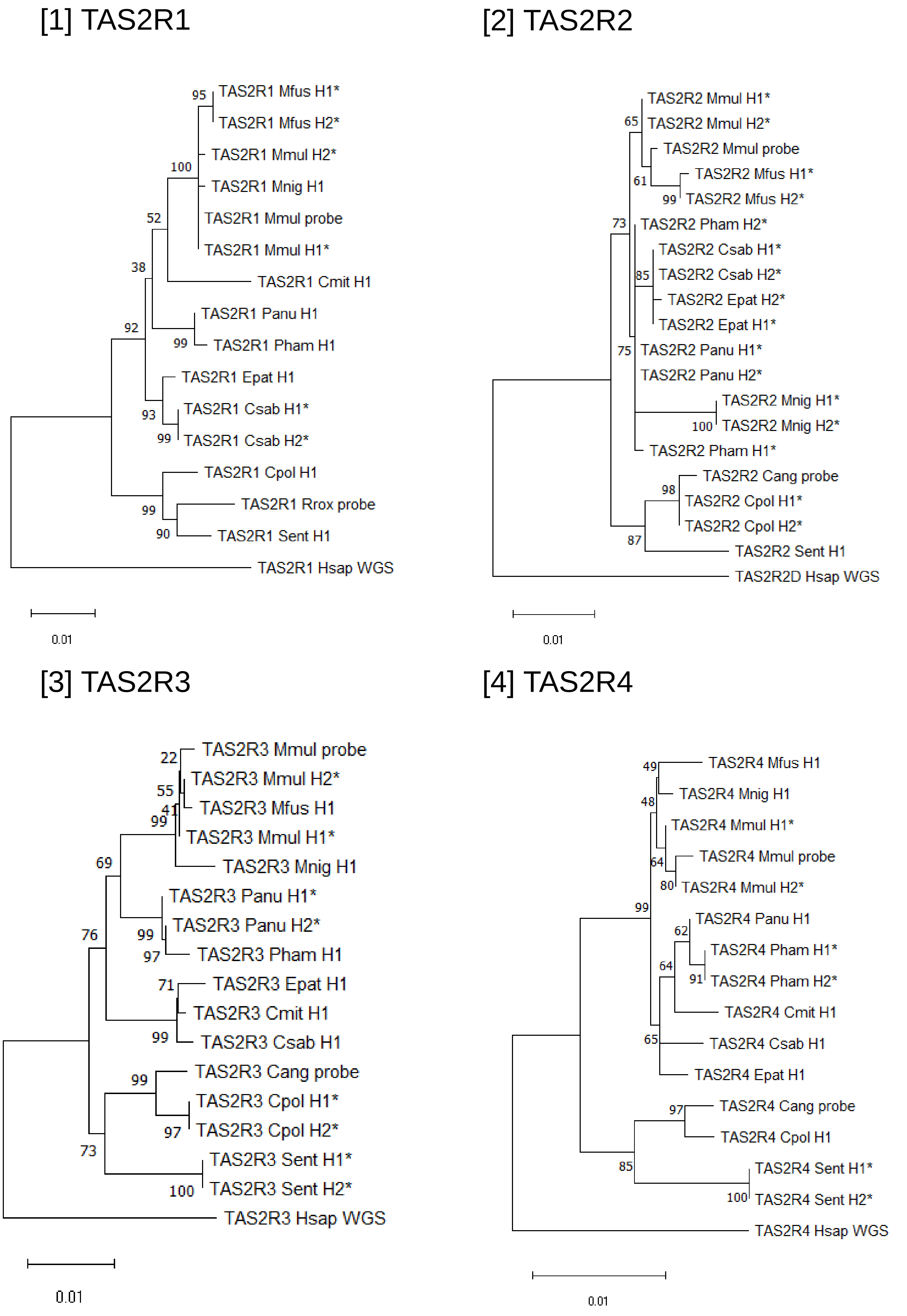


Hou et al. Fig. S4

## [5] TAS2R5

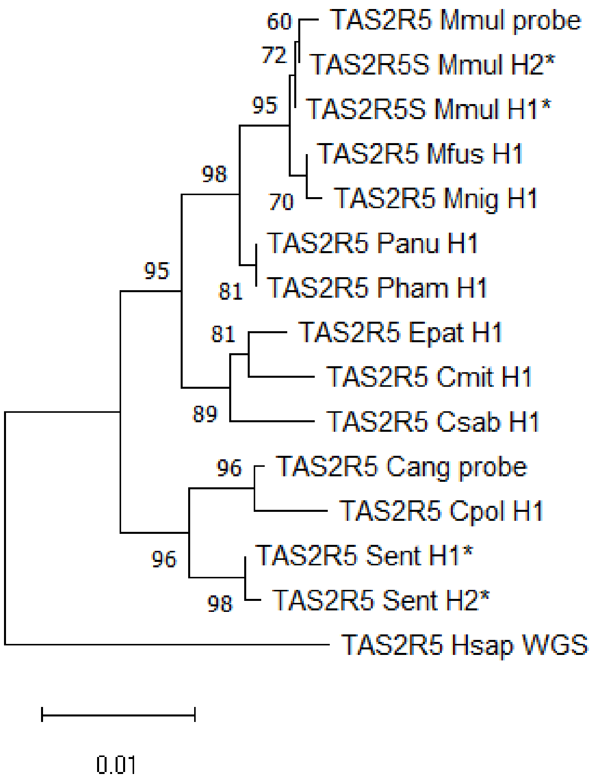


## [6] TAS2R7

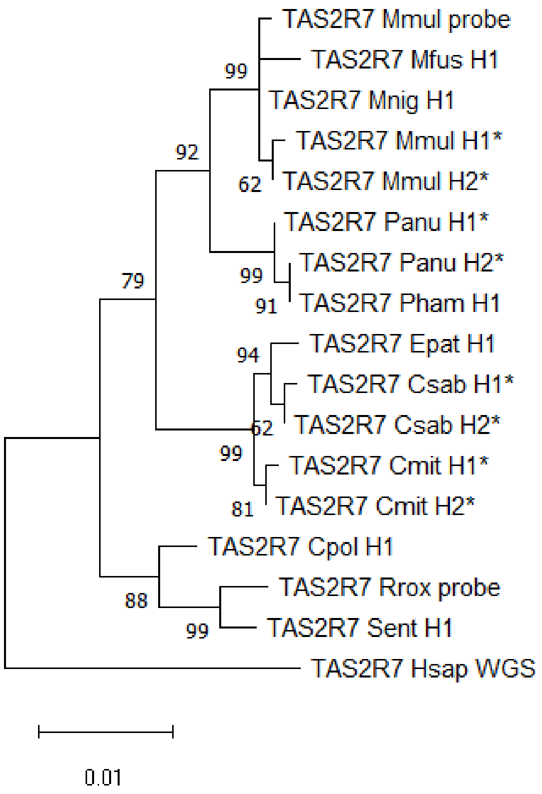


## [7] TAS2R8

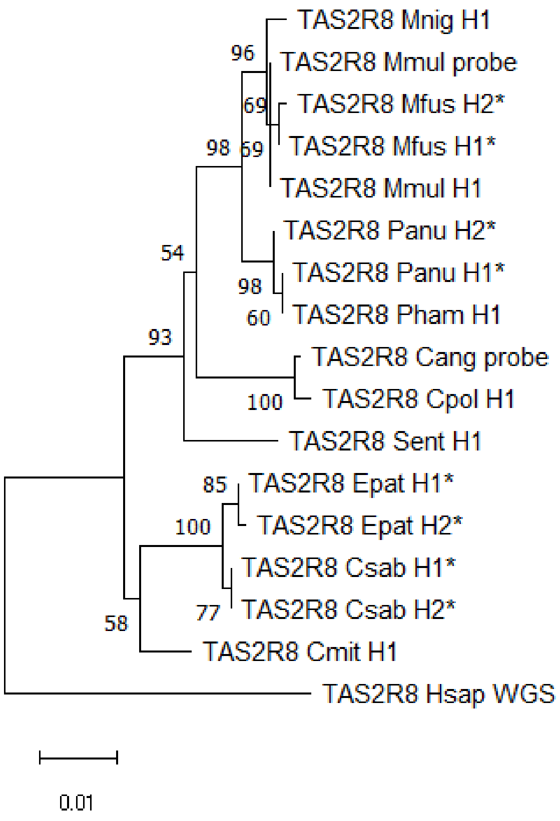


## [8] TAS2R9

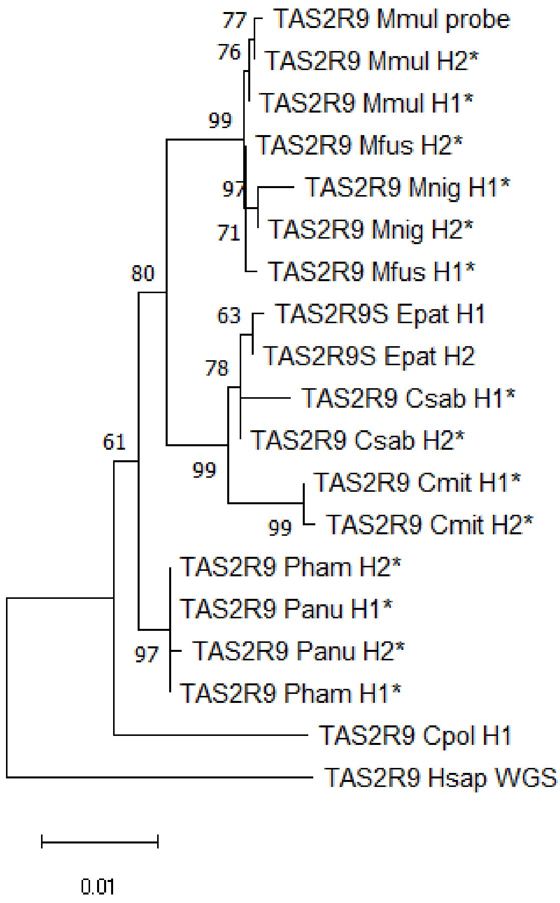


Hou et al. Fig. S4

# [9] TAS2R10-1 & -2

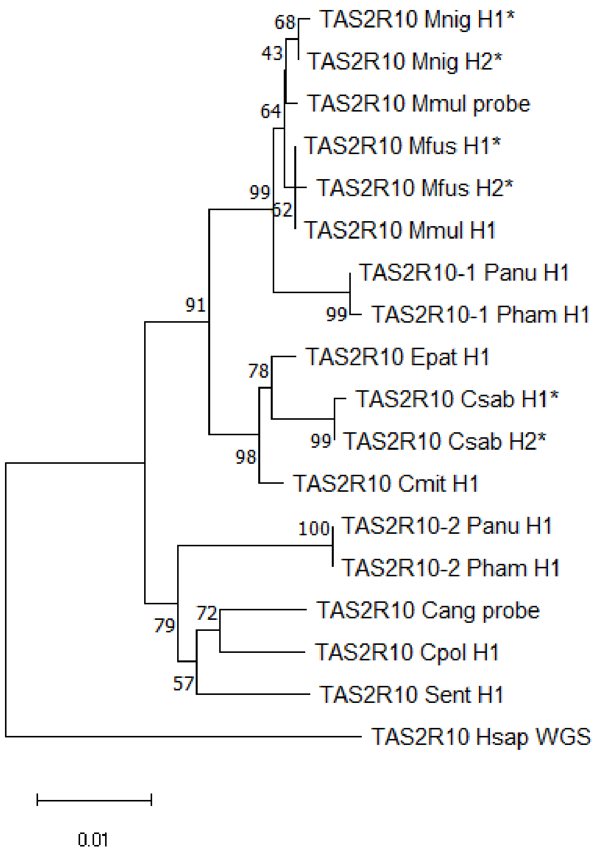


# [10] TAS2R13

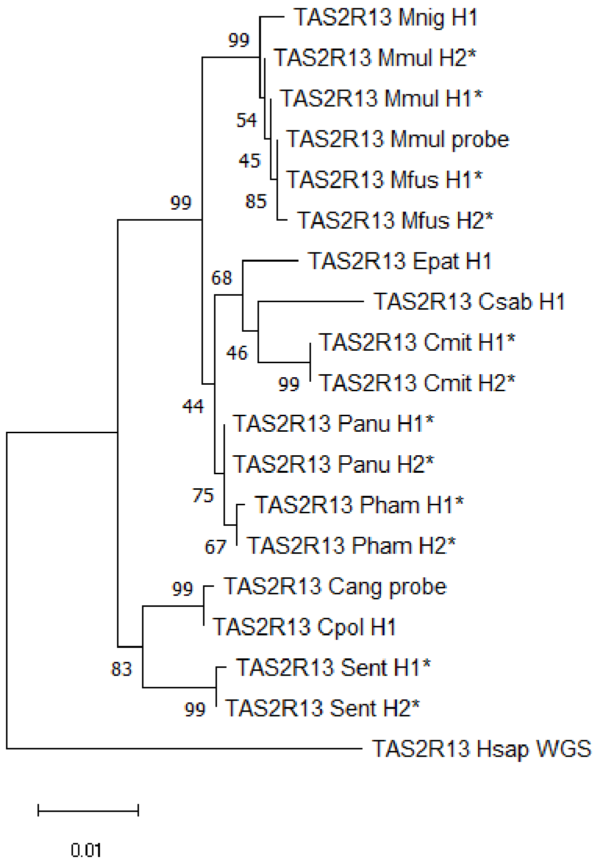


# [11] TAS2R14

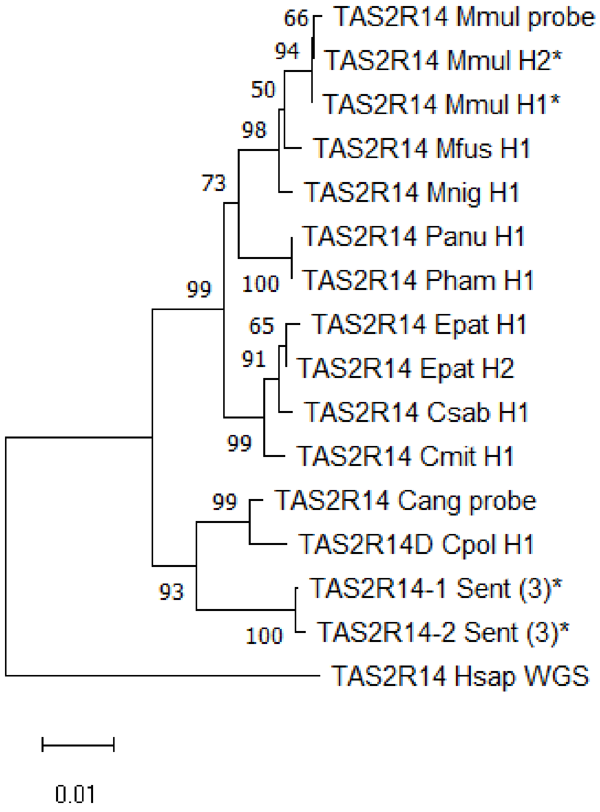


# [12] TAS2R16

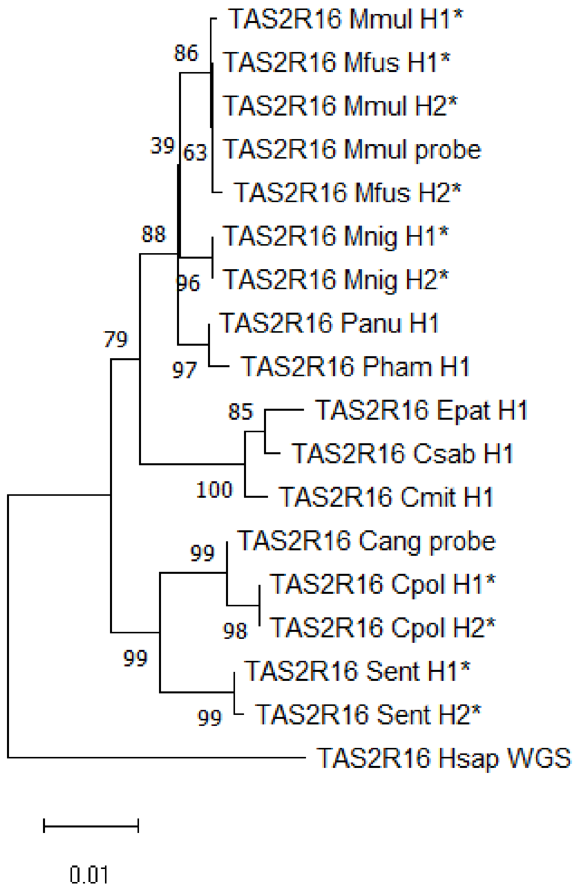


Hou et al. Fig. S4

## [13] TAS2R20

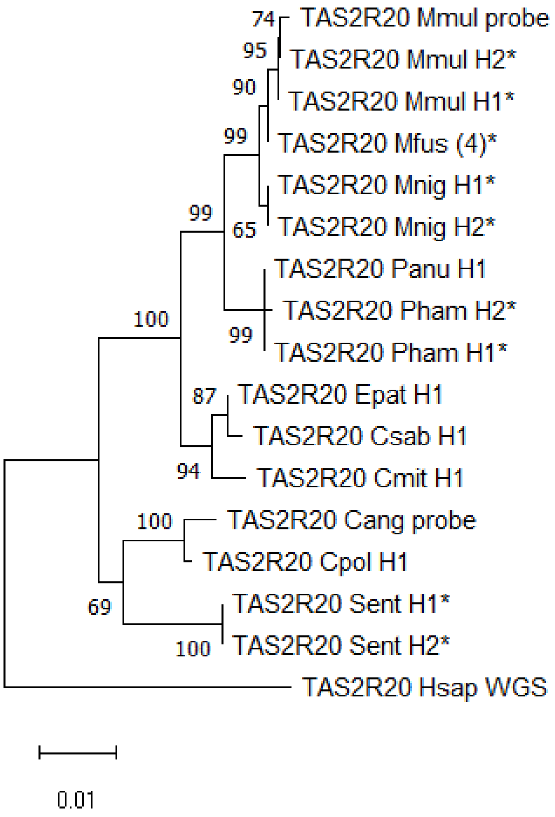


## [14] TAS2R38

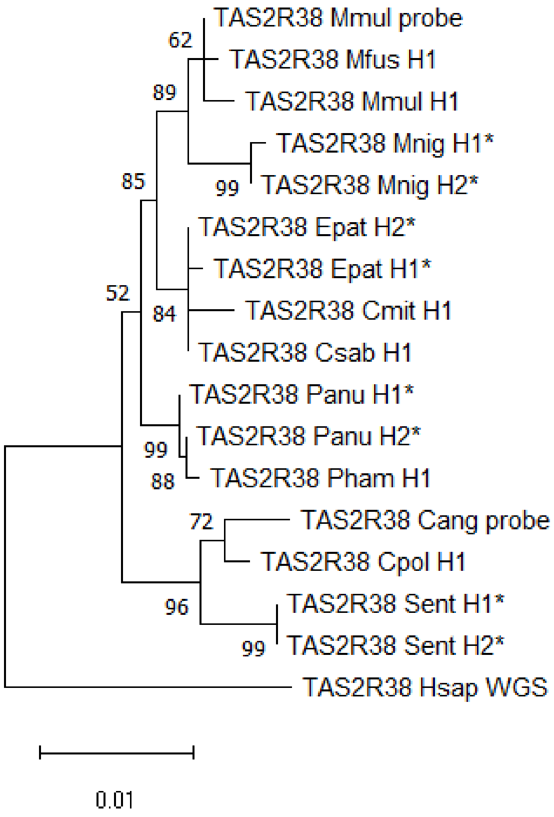


## [15] TAS2R39

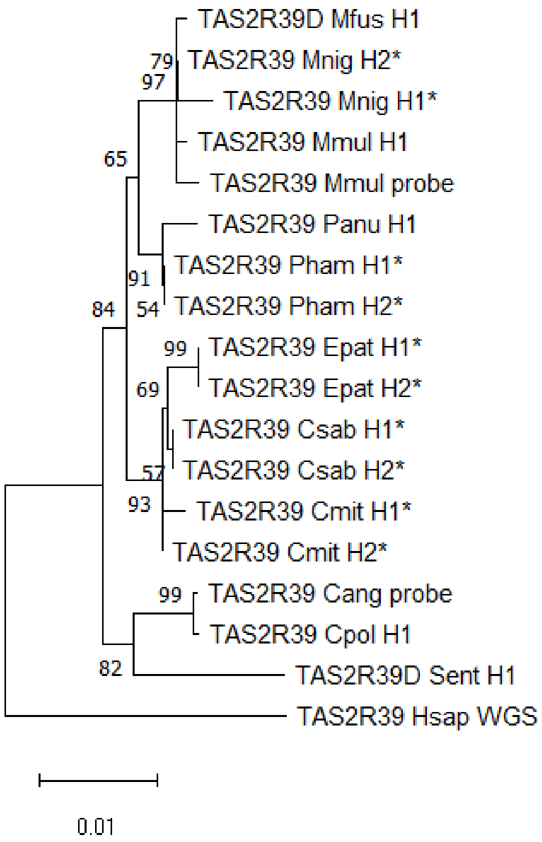


## [16] TAS2R40

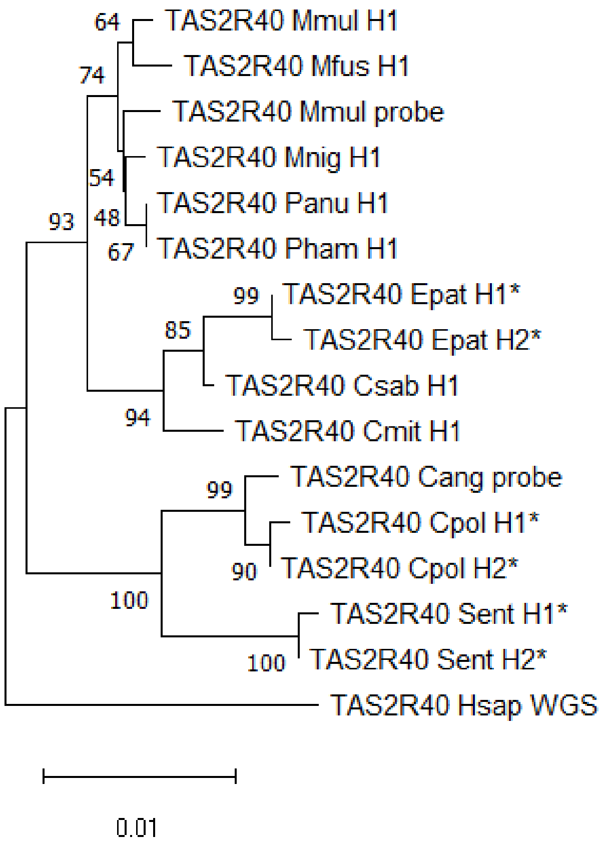


Hou et al. Fig. S4

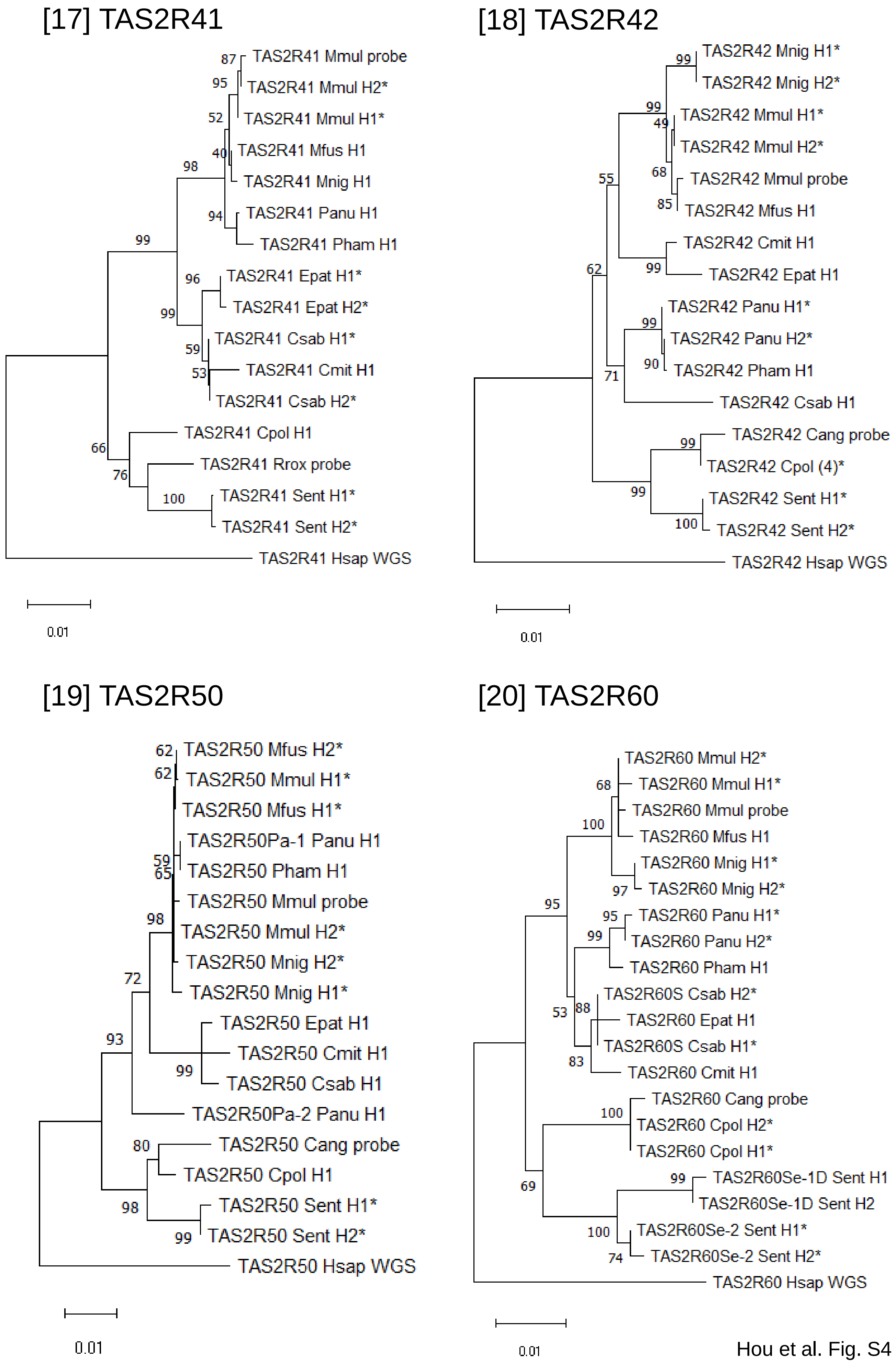


Hou et al. Fig. S4

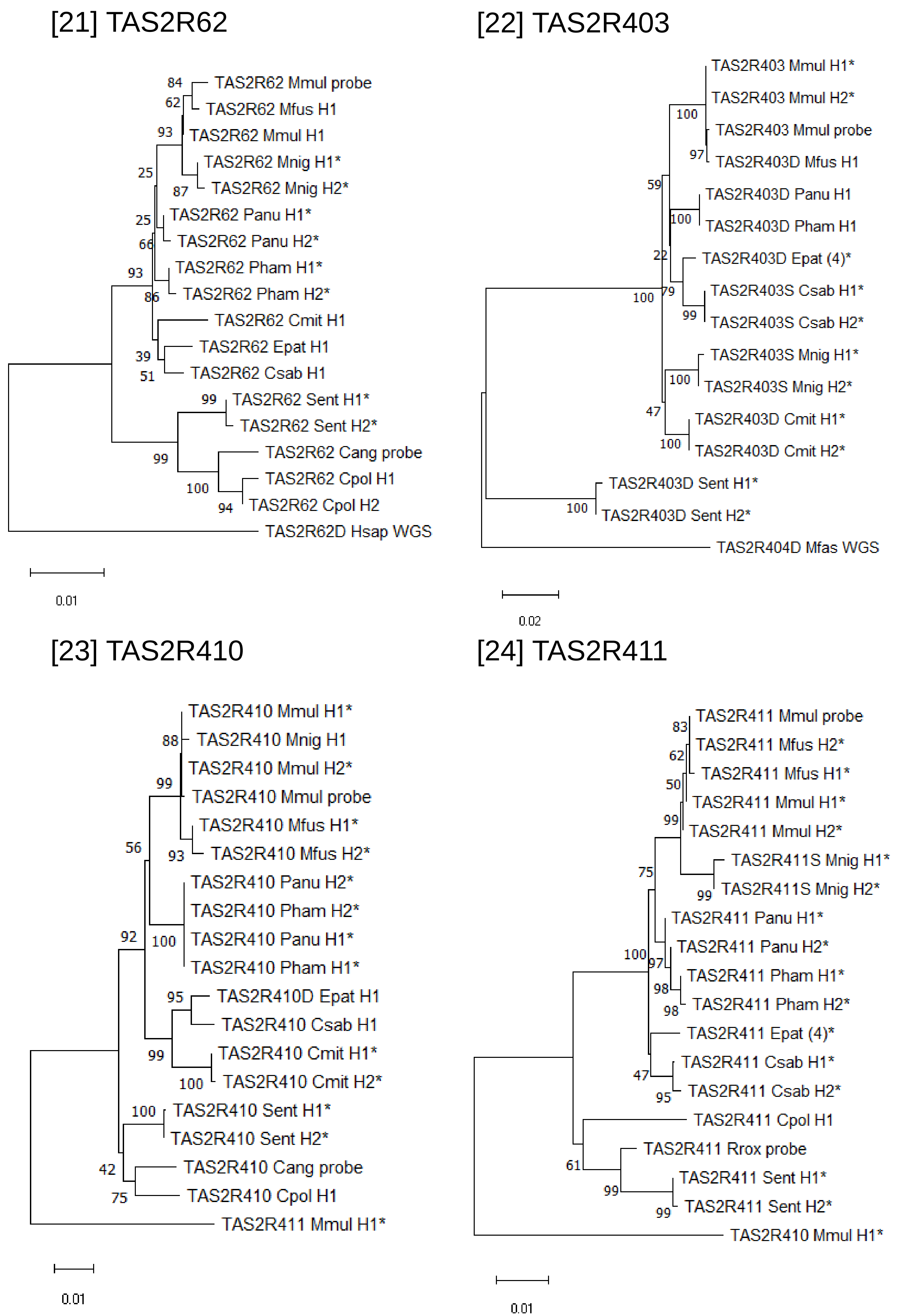


Hou et al. Fig. S4

## [25] TAS2R412

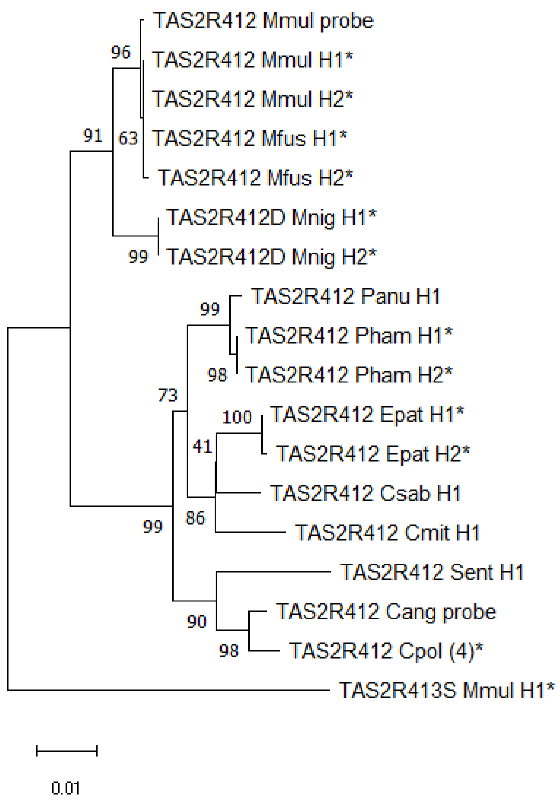


## [26] TAS2R413-1 & -2

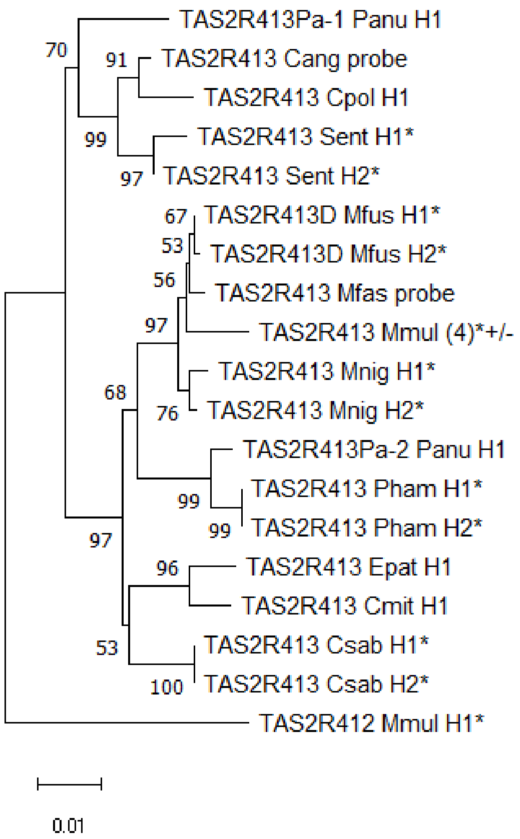


## [27] TAS2R414

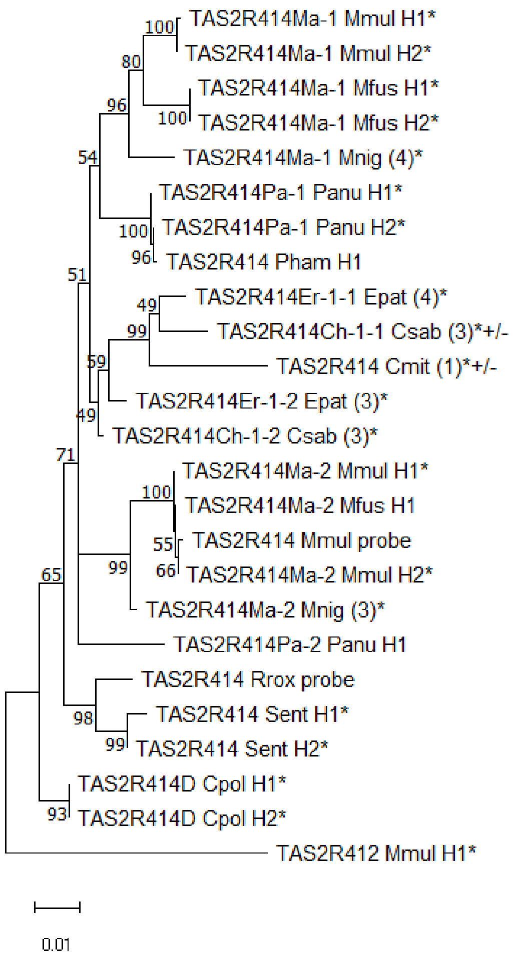


## [28] TAS2R415

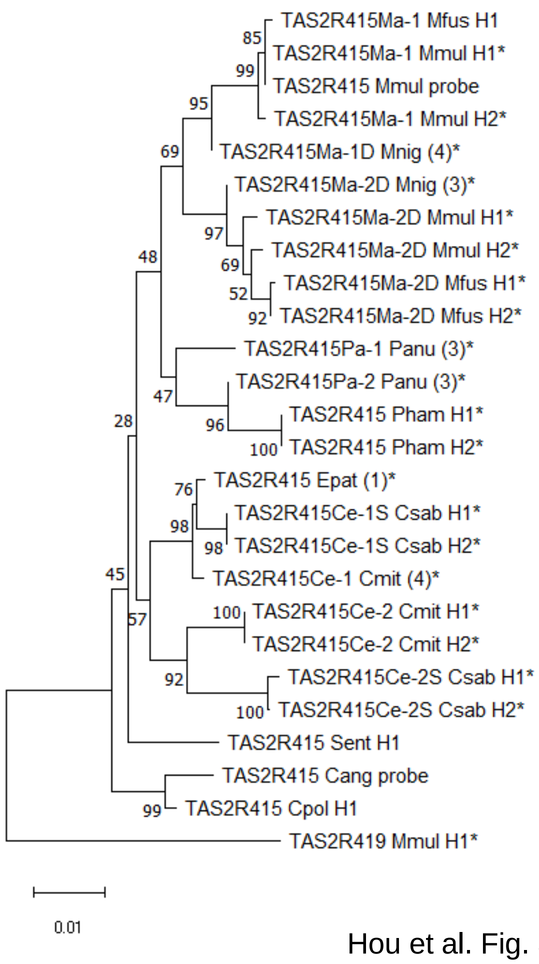


Hou et al. Fig. S4

## [29] TAS2R418

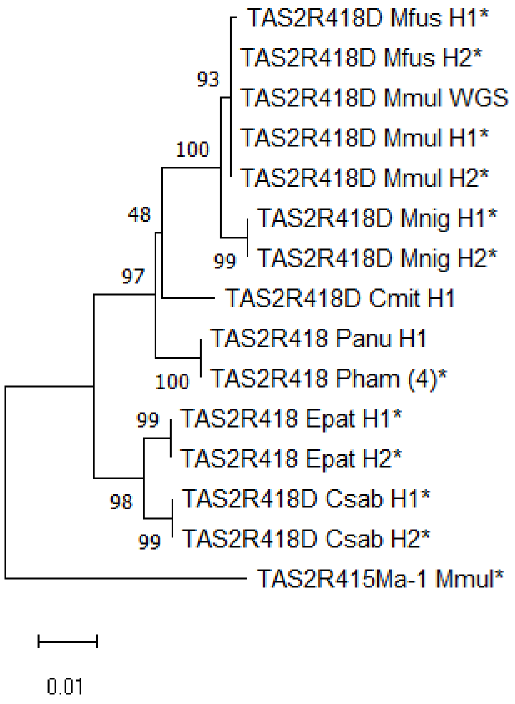


## [30] TAS2R419

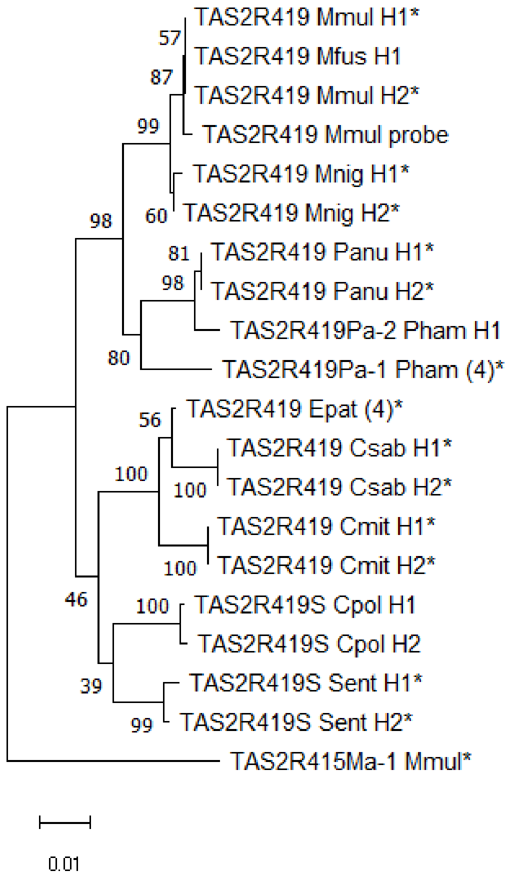


Hou et al. Fig. S4

**Fig. S4.** Phylogenetic trees reconstructed for each of the ancestral-cercopithecid *TAS2R* genes. [1] *TAS2R1*, [2] *TAS2R2*, [3] *TAS2R3*, [4] *TAS2R4*, [5] *TAS2R5*, [6] *TAS2R7*, [7] *TAS2R8*, [8] *TAS2R9*, [9] *TAS2R10-1* and *TAS2R10-2*, [10] *TAS2R13*, [11] *TAS2R14*, [12] *TAS2R16*, [13] *TAS2R20*, [14] *TAS2R38*, [15] *TAS2R39*, [16] *TAS2R40*, [17] *TAS2R41*, [18] *TAS2R42*, [19] *TAS2R50*, [20] *TAS2R60*, [21] *TAS2R62*, [22] *TAS2R403*, [23] *TAS2R410*, [24] *TAS2R411*, [25] *TAS2R412*, [26] *TAS2R413-1* and *TAS2R413-2*, [27] *TAS2R414*, [28] *TAS2R415*, [29] *TAS2R418*, and [30] *TAS2R419*. Note that among the 32 ancestral-cercopithecid *TAS2R* genes, *TAS2R10-1* and *TAS2R10-2* are included in the tree [9] and *TAS2R413-1* and *TAS2R413-2* are in the tree [26]. A sequence name consists of the gene name followed by the species name abbreviation (e.g., TAS2R1 Mmul: see Table 1 and Table S1-2 for abbreviation: human is abbreviated to Hsap). For the single-locus genes, both allele sequences (labeled "H1" or "H2" after sequence names) are used in the trees if the two sequences are different whereas one sequence (labeled "H1") is used if two allele sequences are identical. For Categories (1) and (3) duplicated / multiplicated genes and Category (4) genes with high SNP density but not with high sequencing depth, a congregated sequence is used and labeled "(1)", "(3)", and "(4)", respectively, after the sequence name. Sequences containing IUPAC degenerate nucleotide codes are marked with asterisk (*). Disrupted sequences are suffixed with "D" after the gene name. Segregating pseudogenes are suffixed with "S" after the gene name. The congregated sequences for which both intact and disrupted sequences were inferred (Table S7) are labeled +/- after the sequence names. The sequences used as probes of the targeted capture are labeled "probe" after the sequence names. Regarding *TAS2R418* [29] for which probes were not prepared, a disrupted sequence from rhesus macaque WGA database (Mmul_10) is included (TAS2R418D Mmul WGA). For the non-*TAS2R405* group genes ([1] ~ [22]), their human orthologs are used as an outgroup except for *TAS2R403* [22]. For *TAS2R403*, a disrupted *TAS2R404* gene from the crab-eating macaque WGA database MFA1912RKSv2 is used (TAS2R404D Mfas) because *TAS2R403* arose from *TAS2R409* at the cercopithecid common ancestor together with *TAS2R404* which was subsequently disrupted at the cercopithecid common ancestor (Hayakawa et al. 2014). For the *TAS2R405* group genes ([23] ~ [30]), the rhesus macaque gene from the nearest *TAS2R405* group gene is used as an outgroup. The bootstrap probabilities after 1000

replication are given to each node. The scale bar indicates one nucleotide substitution per 100 sites.
